# Remobilization of dormant carbon from Siberian-Arctic permafrost during three past warming events

**DOI:** 10.1126/sciadv.abb6546

**Published:** 2020-10-16

**Authors:** Jannik Martens, Birgit Wild, Francesco Muschitiello, Matt O’Regan, Martin Jakobsson, Igor Semiletov, Oleg V. Dudarev, Örjan Gustafsson

**Affiliations:** 1 Department of Environmental Science, Stockholm University, 11418 Stockholm, Sweden.; 2 Bolin Centre for Climate Research, Stockholm University, 10691 Stockholm, Sweden.; 3 Department of Geography, University of Cambridge, CB2 3EN Cambridge, UK.; 4 NORCE Norwegian Research Centre, 5007 Bergen, Norway.; 5 Department of Geological Sciences, Stockholm University, 10691 Stockholm, Sweden.; 6 Pacific Oceanological Institute FEB RAS Vladivostok, 690041 Vladivostok, Russia.; 7 Institute of Natural Resources, Tomsk Polytechnic University, 634050 Tomsk, Russia.; 8 International Arctic Research Center, University of Alaska Fairbanks, Fairbanks, 99775 AK, USA.

## Abstract

Carbon cycle models suggest that past warming events in the Arctic may have caused large-scale permafrost thaw and carbon remobilization, thus affecting atmospheric CO_2_ levels. However, observational records are sparse, preventing spatially extensive and time-continuous reconstructions of permafrost carbon release during the late Pleistocene and early Holocene. Using carbon isotopes and biomarkers, we demonstrate that the three most recent warming events recorded in Greenland ice cores—(i) Dansgaard-Oeschger event 3 (~28 ka B.P.), (ii) Bølling-Allerød (14.7 to 12.9 ka B.P.), and (iii) early Holocene (~11.7 ka B.P.)—caused massive remobilization and carbon degradation from permafrost across northeast Siberia. This amplified permafrost carbon release by one order of magnitude, particularly during the last deglaciation when global sea-level rise caused rapid flooding of the land area thereafter constituting the vast East Siberian Arctic Shelf. Demonstration of past warming-induced release of permafrost carbon provides a benchmark for the sensitivity of these large carbon pools to changing climate.

## Teaser

Arctic Ocean sediments reveal permafrost thaw and carbon release during three large warming events of the past 27,000 years.

## INTRODUCTION

Anthropogenic climate change has, over recent decades, resulted in rapid warming of the Arctic region (*1*). One consequence is thawing of permafrost (*1*), which currently holds a stock of organic carbon (OC) estimated to be 1300 Pg (*2*), about twice the inventory of atmospheric CO_2_. Permafrost thaw includes gradual changes, such as deepening of the seasonally thawed surface soil (i.e., active layer) and loss of permafrost extent, as well as rapid thaw by thermal landscape collapse, thermokarst, and erosion of coastlines and river banks (*3*, *4*). By exposing previously freeze-locked OC to microbial degradation, these processes may cause a permafrost carbon–climate feedback that reinforces anthropogenic emissions of greenhouse gases (e.g., CO_2_ and CH_4_) (*1*). Anticipating the strength and timing of this nonlinear carbon cycle response is a major challenge in climate change science. Past warming events may provide our best opportunity to learn about the response behavior of the massive freeze-locked permafrost OC pool to a warming climate.

**Fig. 1:**
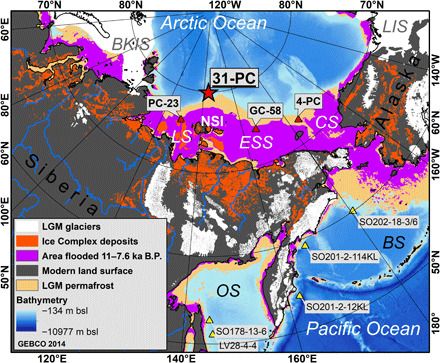
Paleoenvironment of the LGM (20 ka B.P.) with the location of core 31-PC (red star; SWERUS-C3 expedition 2014). Bathymetry of the Arctic and Northern Pacific Ocean are from GEBCO 2014. The beige shade marks the additional-to-present permafrost-covered land surface during the LGM, purple marks the area flooded during the EH (11 to 7.6 ka B.P.; based on shelf bathymetry and global sea-level rise) (*5*, *31*), and dark gray marks the modern land surface. The orange color indicates the distribution of ICDs in periglacial regions of Siberia and Alaska (*10*). LIS, Laurentide Ice Sheet; BS, Bering Sea; CS, Chukchi Sea; ESS, East Siberian Sea; LS, Laptev Sea; BKIS, Barents-Kara Sea Ice Sheet; OS, Sea of Okhotsk; NSI, New Siberian Islands. Red triangles indicate the location of cores described in previous studies on the Arctic shelves, the 4-PC (*13*), GC-58 (*17*), and PC-23 (*11*). Yellow triangles indicate the location of cores located in the Pacific realm; the cores SO202-18-3/6, SO201-2-114KL, and SO201-2-12KL in the Bering Sea (*18*); as well as the cores SO178-13-6 and LV28-4-4 in the Sea of Okhotsk (*14*).

The last deglaciation—here defined as the time period between the Last Glacial Maximum [LGM; 26 to 20 thousand years before the present (ka B.P.)] (*5*) and the early Holocene climate optimum (EH; 10 to 8 ka B.P.)—marked a period of profound climate change. This period witnessed rapid warming by ~3.5°C in the Northern Hemisphere (*6*), the demise of Arctic ice sheets resulting in a global sea-level rise of 134 m (*5*), and a rise of atmospheric CO_2_ concentrations by 80 ppmv (parts per million by volume) (*7*). It is also suggested to have caused broad reorganization of OC pools in Arctic permafrost (*8*). This particularly affected the ice- and OC-rich Ice Complex deposits (ICD; also referred to as Yedoma), which developed during the Pleistocene in nonglaciated Arctic regions (*9*). During the LGM global sea-level low stand of −134 m, up to 40-m-thick ICD accumulated in northeastern Siberia likely over large areas that now make up the world’s largest conventional shelf—the East Siberian Arctic Shelf (ESAS) (*8*). The postglacial sea-level rise and flooding of what became the ESAS is thought to have removed about 220 to 260 Pg of OC (*8*, *10*), leaving 400 Pg of total above-sea ICD-OC in near-coastal areas in Siberia and Alaska (Fig. 1) (*10*).

During the last deglaciation, large-scale permafrost thawing, including thaw of inland permafrost (*11*, *12*) and flooding with resultant thermo-erosion of ICD on the then-exposed ESAS (*13*), is suggested to have liberated CO_2_ by biodegradation of the thawed organic matter and thus have contributed to an increase in atmospheric CO_2_ concentrations by 80 ppmv or 200 Pg carbon (*14*–*16*). Another important source of CO_2_ was outgassing of deep ocean water by changing oceanic circulation at that time (*7*). However, atmospheric archives suggest that the deglacial rise in CO_2_ was caused by ^13^C- and ^14^C-depleted carbon, and oceanic CO_2_ alone may not fully explain this isotopic anomaly (*15*). Hence, inundation of the Arctic Ocean shelves and degradation of permafrost OC, followed by emissions of ^14^C-depleted CO_2_, may also have contributed to part of the rise in atmospheric CO_2_ (*14*). A few spatially limited and noncontinuous observational studies have confirmed with radiocarbon dating that the deglacial sea-level rise remobilized old OC, likely due to coastal retreat of permafrost (*13*, *17*). Two studies from the northeast Pacific suggest that this also happened outside the Arctic Ocean during shelf inundation in the Bering Sea and the Sea of Okhotsk (Fig. 1) (*14*, *18*). There is, however, no single continuous archive that explores the remobilization of OC from Arctic permafrost during the last deglaciation, leaving both the magnitude and the onset and end of this hypothetically massive permafrost OC remobilization largely unconstrained.

Here, we present the first continuous record of permafrost OC remobilization from the Siberian-Arctic continental margin from before the LGM (27 ka B.P.) to the preindustrial. Carbon isotopes (Δ^14^C and δ^13^C) and terrigenous biomarkers [lignin phenols; high–molecular weight (HMW) *n*-alkanes and *n*-alkanoic acids] were analyzed in the marine sediment core SWERUS-L2-31-PC1 (below abbreviated 31-PC), retrieved in 2014 during the SWERUS-C3 expedition (Swedish-Russian-U.S. East Siberian Arctic Ocean Investigation of Climate-Cryosphere-Carbon Interactions) onboard the Swedish icebreaker *Oden*. The 8-m-long core, with a basal age of 27.3 + 2.3/−1.4 ka B.P. (*19*), was recovered from the Southern Lomonosov Ridge in the Arctic Ocean north of the New Siberian Islands (Fig. 1). In contrast to the previous studies in the Arctic Ocean, this archive is uniquely located to record inputs from seaward-transported carbon from a broad catchment area in the East Siberian region and provides a record across the full deglaciation period. To distinguish between the two dominating thaw and permafrost-carbon remobilization processes operating today, which are gradual permafrost active layer thaw versus abrupt thermal collapse of ICD (*20*), this study used a statistical mixing model of carbon isotopes (*21*) to quantitatively apportion the relative source contributions to the terrestrial OC that was released. Accordingly, the mixing model thus includes two terrigenous end members, which are permafrost active layer (Δ^14^C-OC of −197.5 ± 148.3‰; δ^13^C-OC of −26.4 ± 0.8‰) (*22*) and ICD (Δ^14^C-OC of −962 ± 61‰; δ^13^C-OC of −26.3 ± 0.7‰) (*22*, *23*) using published ^13^C and ^14^C data of permafrost deposits and soils in Siberia, as well as a marine OC end member based on measurements of marine phytoplankton (Δ^14^C-OC of −50 ± 12‰; δ^13^C-OC of −21.0 ± 2.6‰) (*13*). This study provides the first evidence for permafrost carbon export across the LGM and through the Holocene, elucidating the importance of permafrost for atmospheric CO_2_ and illustrating the sensitivity of permafrost OC pools to polar warming.

## RESULTS

### Permafrost carbon release during Dansgaard-Oeschger event 3

**Fig. 2:**
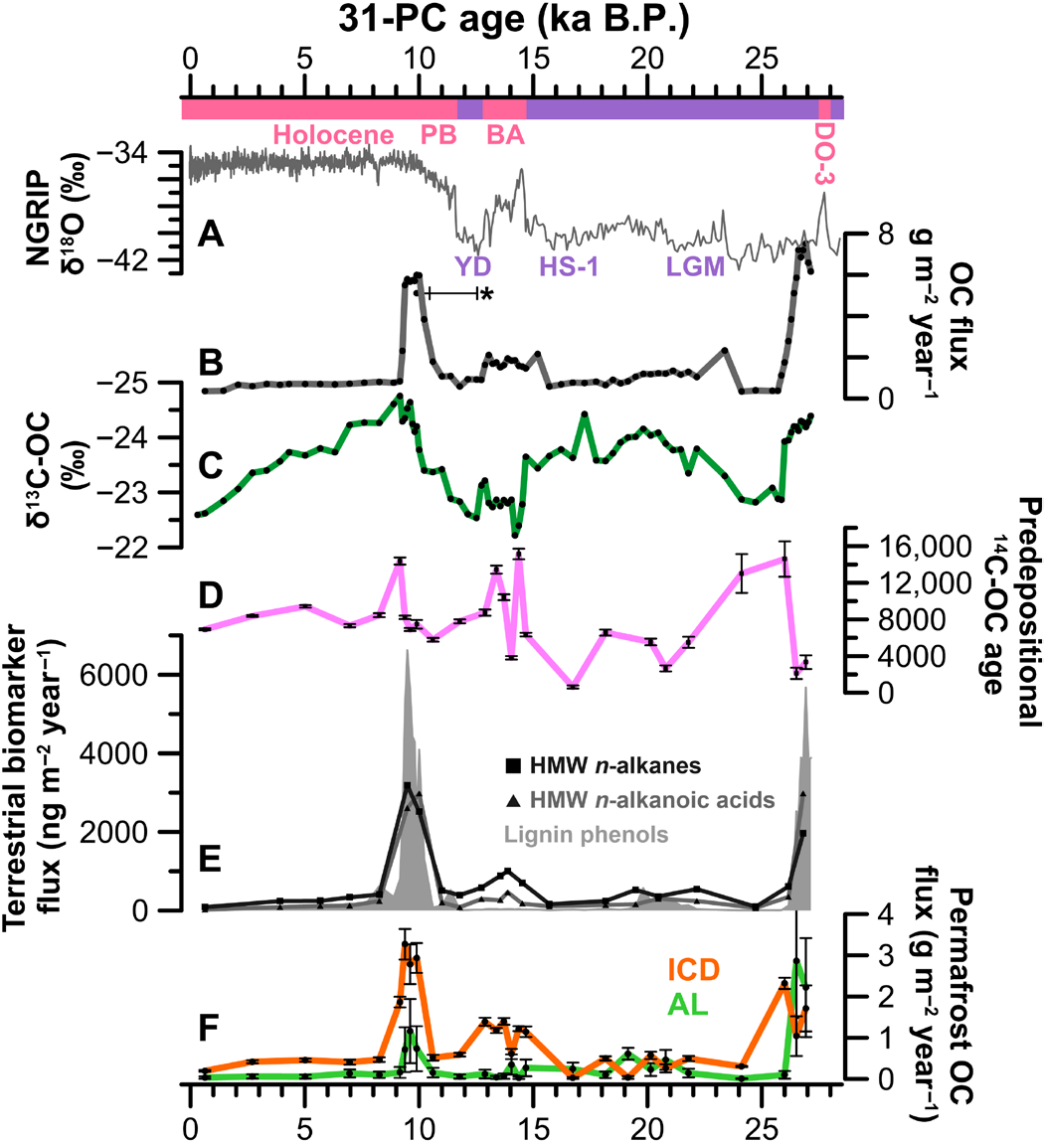
Bulk carbon characteristics and biomarkers in sediments of core 31-PC over the past 27 ka B.P., which covers the Dansgaard-Oeschger event 3 (DO-3), the cold Last Glacial Maximum (LGM), the Heinrich Stadial 1 (HS-1), the Bølling-Allerød (BA) warm interstadial, the cold Younger Dryas (YD) stadial, and the warm Holocene/Interglacial [including the EH Preboreal (PB)]. The curves show (**A**) the North Greenland Ice Core Project (NGRIP) δ^18^O record (*39*), (**B**) the total OC flux to the 31-PC location (g m^−2^ year^−1^), (**C**) δ^13^C of OC, and (**D**) the predepositional ^14^C age of OC. It further shows (**E**) terrestrial biomarker fluxes of lignin phenols, HMW *n*-alkanes and *n*-alkanoic acids (μg m^−2^ year^−1^), as well as (**F**) fractions of the total OC flux (g m^−2^ year^−1^) and the 1σ uncertainties based on statistical source apportionment of OC mobilized from the two permafrost pools ICDs and permafrost active layer (AL). *The range bar indicates extent of possible delay in arrival of the PF-C signal from EH warming, due to the large increase of cross-shelf transport times (text S2) (*40*).

Although permafrost is often thought to broadly function as an OC sink during glacial periods, 31-PC reveals a strong peak of released terrestrial OC during the early stage of the 27 ka record, before the onset of the LGM (Fig. 2). Statistical source apportionment based on δ^13^C-OC and Δ^14^C-OC suggests that most of this OC was terrestrial and predominantly originated from permafrost active layer (34 to 40% of the OC). The high flux of terrigenous OC indicated by the dual-isotope source apportionment (~3.9 g m^−2^ year^−1^) is supported by terrigenous biomarkers, which were consistently higher in this period compared to the subsequent LGM (lignin phenols >10, HMW *n*-alkanes ~4, HMW *n*-alkanoic acids ~8; Fig. 2).

**Fig. 3:**
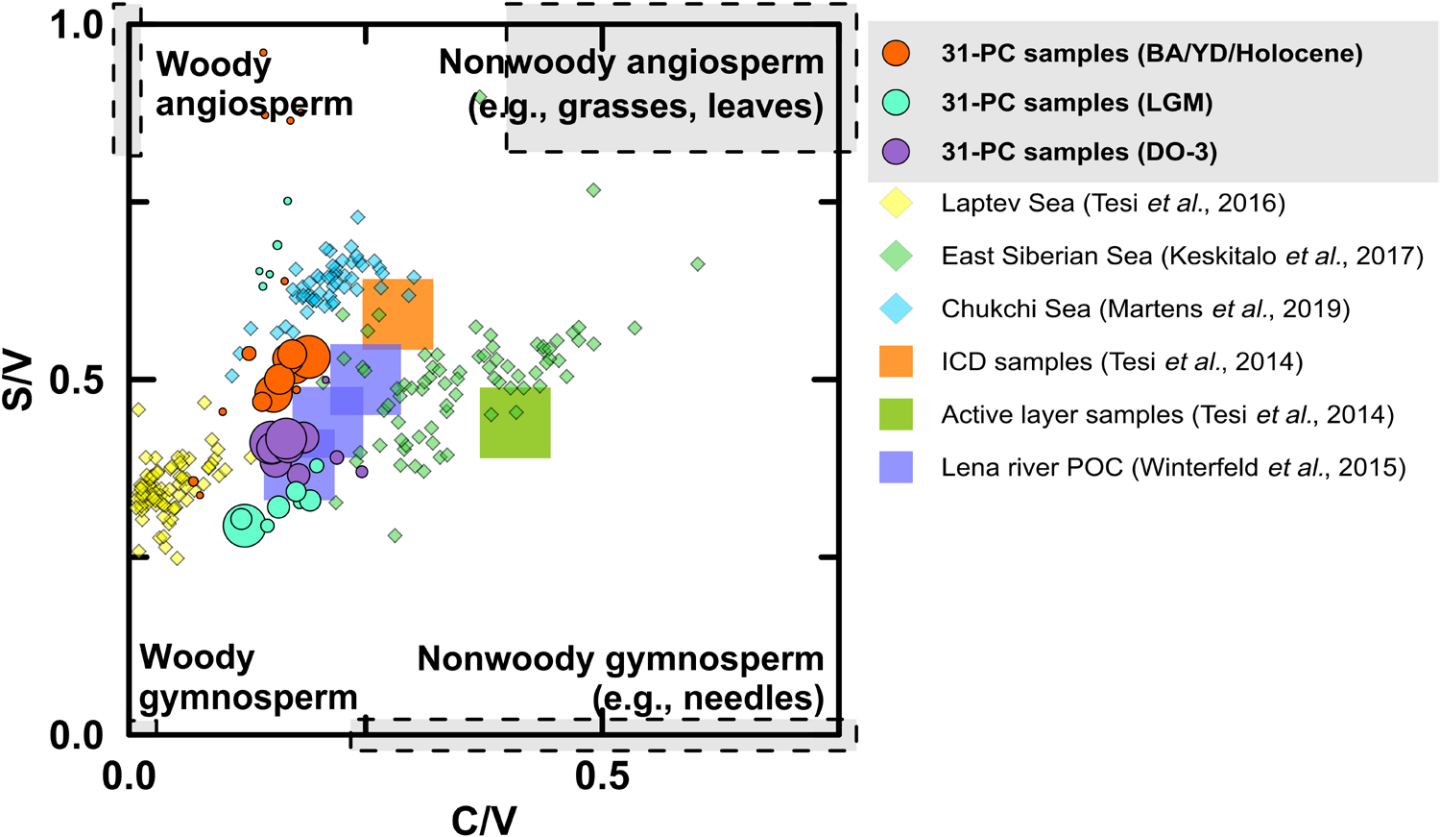
Lignin source diagnostics of terrigenous material in the 31-PC (80 samples) compared with previously studied sediment cores from the Siberian Shelf 4-PC (*13*), GC-58 (*17*), and PC-23 (*11*), as well as coastal ICD, AL permafrost (*32*), and Lena river particulate OC (*25*). The size of the 31-PC circles is proportional to the magnitude of the respective lignin flux (0.01 to 6.6 mg year^−1^ m^−2^). The abundance ratio of syringyl over vanillyl (S/V) indicates contributions of angiosperm compared to gymnosperm plants, and the ratio between cinnamyl and vanillyl (C/V) distinguishes between woody (e.g., stems) and nonwoody (e.g. leaves) plant tissues (*24*).

A provenance proxy based on lignin phenols provides additional information about the source of the terrigenous OC. The low ratio of syringyl over vanillyl phenols (S/V) indicates a strong contribution of gymnosperm lignin tissues (*24*) in sediments deposited between 27 and 26 ka B.P. (S/V, 0.41 ± 0.04; *n* = 11). This resembles the fingerprint of end members from more southern biomes hosting gymnosperm forests, similar to contemporary suspended fluvial material in the Lena river (~0.4) that drains these forests in central Siberia (Fig. 3) (*25*). Hence, comparatively shallow active layer material mobilized in central Siberia and transported by rivers is the most likely source of the large amounts of OC deposited on the southern Lomonosov Ridge between 27 and 26 ka B.P. The limited contribution from coastal thermo-erosion of permafrost at that time is consistent with global sea-level regression during the buildup to the LGM (*5*).

Biomarker degradation proxies suggest that terrigenous OC deposited 27 to 26 ka B.P. was less degraded than terrigenous OC deposited during the LGM, which indicates rapid remobilization and fluvial transport of the organic matter. This is shown by the ratio of 3,5-dihydrobenzoic acid relative to vanillyl (3,5-Bd/V), reflecting the removal of the more vulnerable vanillyl with increasing degradation of OC starting from 27 ka B.P. (0.6 to 1.6; fig. S2) toward the LGM (>5), as well as by the preferential decomposition of HMW *n*-alkanoic acids relative to HMW *n*-alkanes (1.5 versus 0.4 to 0.7). Taken together, the degradation pattern reveals transport of little-degraded plant material from distant sources, likely located in central Siberia, which suggests a period of accelerated discharge or a pulse of terrigenous OC to the Arctic Ocean.

Dansgaard-Oeschger (DO) events represent periods of abrupt warming and strong hydrological change during the last glacial period (*26*). While DO events are known to affect the global climate and carbon cycle, only a few records are able to attribute environmental change in Siberia to the effects of DO events. A study from Lake Baikal suggested that profound changes in precipitation triggered soil erosion events in the lake catchment synchronous to DO and Heinrich events in the North Atlantic region (*27*). It is likely that the terrigenous OC peak in 31-PC resulted from surface permafrost thawing in response to abrupt climate warming and increased precipitation during the DO-3 event peaking around 28 ka B.P. (Fig. 2) (*26*). There is greater uncertainty around this event compared to the two more recent warming events detailed below due to a combination of (i) the lack of recovered core material to document also the onset of the DO-3 event in the 31-PC record, (ii) uncertainty in the 31-PC age-depth model for the core base of 27.3 + 2.3/−1.4 ka B.P. (90% uncertainty range; fig. S1) (*19*), and (iii) the possible delay between inland permafrost thaw and peak signal arrival at the 31-PC location. This represents, to our knowledge, the first support for permafrost OC release related to DO events during one of the main periods of ICD formation in Siberia (*10*, *23*). Hence, permafrost might have been more vulnerable to abrupt, short-term warming events in this more distant past than previously thought.

### Limited carbon transport during the LGM and the late glacial period

The 31-PC record demonstrates a minimum in terrigenous OC delivery to the southern Lomonosov Ridge near the LGM, between 26 and 20 ka B.P., thereby providing a baseline for assessing dynamics in permafrost OC remobilization during the last deglaciation. Near the onset of the LGM (~26 ka B.P.), the accumulation of OC declined to 1.1 ± 0.6 g m^2^ year^−1^ (*n* = 13). Dual isotope–based source apportionment reveals a terrigenous OC fraction of 65 ± 11% (*n* = 4). Lipid biomarkers (*n*-alkanes and *n*-alkanoic acids) are qualitatively consistent with a strong contribution of terrigenous OC, shown by a predominance of HMW chain length compounds over low–molecular weight (LMW) chain lengths, which is typical for terrigenous organic matter (tables S3 and S5). Fluxes of terrigenous biomarkers (Fig. 2) during the LGM were only one-third (HMW *n*-alkanes) and about one-tenth (for lignin phenols and HMW *n*-alkanoic acids) of fluxes during the preceding DO-3 event. This suggests that cold and dry climatic conditions during the LGM (*28*) disrupted major transport pathways of terrigenous OC to the Arctic Ocean. Previous research stresses that the LGM probably caused large sea-ice expansion with periods of year-round coverage (*19*, *29*) and perhaps even partial ice-shelf coverage in some parts of the Arctic (*30*). A more extensive permanent sea ice cover during the LGM is consistent with low OC accumulation rates in sediments along and off the Siberian continental margin (*19*, *29*).

Despite the cold glacial climate, the predominance of gymnosperm lignin tissues suggests that Arctic rivers likely remained an important gateway for terrigenous OC to reach sediments on the Siberian continental margin. The ratio of S/V indicates higher contribution of gymnosperm lignin tissues (*24*) during the LGM (0.37 ± 0.12; *n* = 7) compared to the DO-3 event (0.41 ± 0.04; *n* = 11; Fig. 3) and even the Holocene (0.70 ± 0.37; *n* = 28).

Biomarker degradation proxies further suggest more extensive degradation of terrigenous OC during the LGM. The 3,5-Bd/V record shows that terrigenous OC deposited during the LGM was strongly degraded (>5; *n* = 12; fig. S2), which is also supported by the depletion of HMW *n*-alkanoic acids relative to HMW *n*-alkanes (0.4 to 0.8 in the LGM versus 1.5 in the DO-3 pulse). Disregarding possible depth uncertainties due to local deviation from the global eustatic mean, the paleo-shoreline was likely less than 100 km from the 31-PC location during the global LGM sea-level low stand. Therefore, the long transport time of terrigenous OC to the 31-PC location, indicated by the degradation state, supports river transport from more southern locations during the LGM. Taken together, the LGM marks a minimum in OC accumulation with most of the OC delivered from distant terrigenous sources, likely with Arctic rivers being an important transport pathway at that time.

### Early deglaciation and carbon remobilization during the Bølling-Allerød warming

The early deglaciation, including the Heinrich stadial 1 (HS-1; also referred to as the Older Dryas), kept permafrost OC pools in a freeze-locked state. Although the HS-1 exhibits the first increase in atmospheric CO_2_ after the LGM at 17.5 ka B.P., results from 31-PC suggest that low terrigenous OC fluxes persisted from the LGM to the HS-1. Our results are largely in line with low accumulation rates of HMW *n*-alkanes in sediment archives from the Bering Sea and the Sea of Okhotsk for HS-1 (fig. S5) (*14*, *18*). This suggests that despite a rise in global sea level by ~35 m (*5*) between 20 and 14.7 ka B.P., cold glacial conditions with steady LGM-like temperatures in the Arctic (*6*) protected permafrost carbon from substantial thaw during this period.

The Bølling-Allerød (BA; 14.7 to 12.9 ka B.P.), a period of rapid atmospheric warming in the Northern Hemisphere (*6*), saw the first substantial increase in terrigenous OC transport relative to the LGM and HS-1. The accumulation rates of HMW *n*-alkanes and HMW *n*-alkanoic acids deposited between 14.7 and 12.9 ka B.P. were twice as large as those recorded before 14.7 ka B.P. (Fig. 2), while fluxes of lignin phenols remained at LGM and HS-1 levels. Furthermore, predepositional ^14^C-OC ages (10.1 ± 4.9 ka; *n* = 5) doubled compared to the organic matter deposited during the HS-1 and LGM (4.5 ± 5.3 ka; *n* = 7), pointing to the release of relatively older OC. Previous studies of sediments in the Sea of Okhotsk (*14*) and the Bering Sea (*18*) reported compound-specific ^14^C ages of terrigenous biomarkers (~10 ka old at the time of deposition) similar to the ^14^C-OC ages observed in this study, suggesting widespread release of strongly preaged terrigenous matter during the BA warm event.

**Fig. 4:**
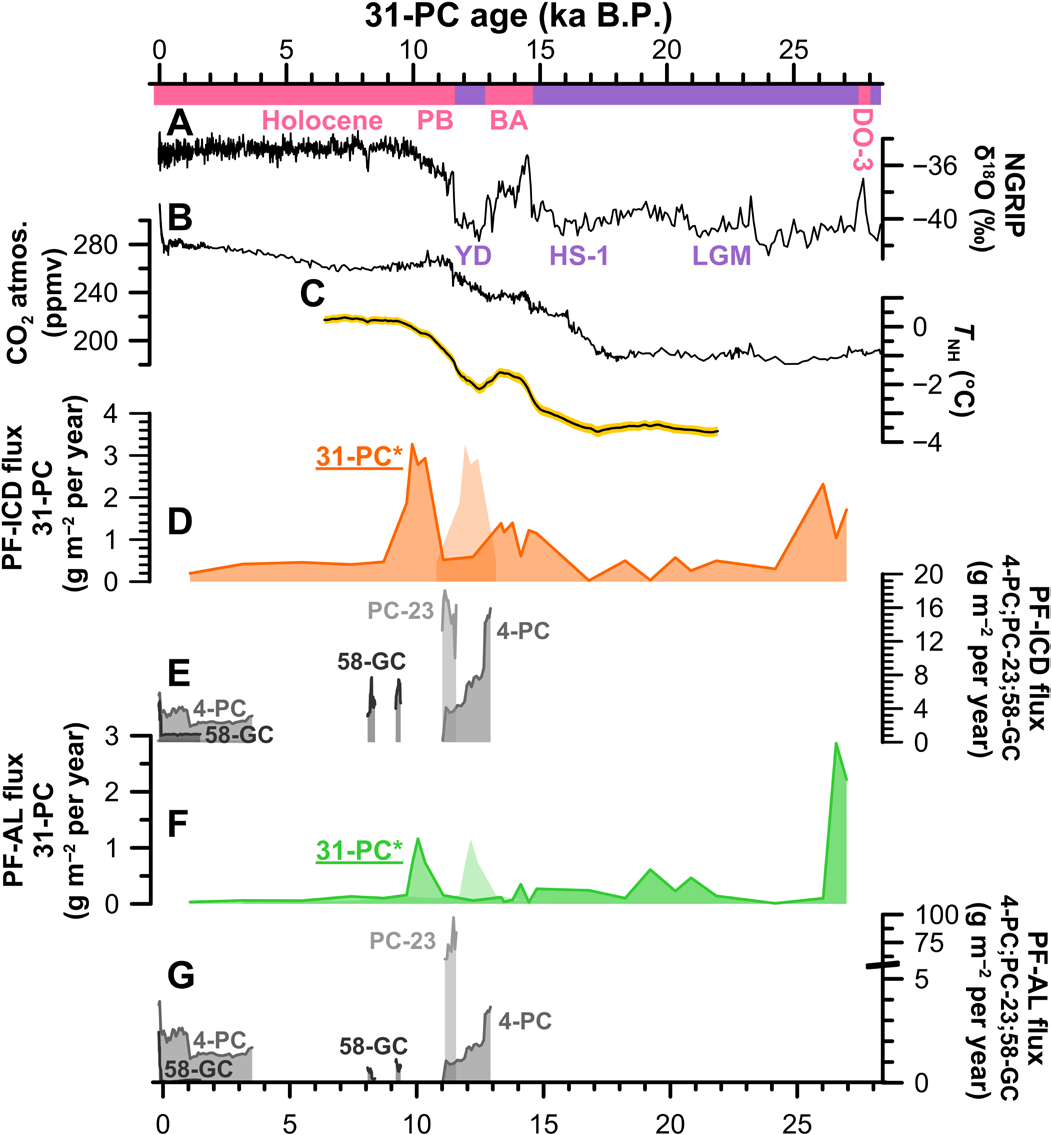
The observational record of permafrost OC fluxes to sediment cores of the ESAS compared with (A) the Greenland ice core δ^18^O record (*39*), (B) the atmospheric CO_2_ concentration (*7*), and (C) Northern Hemisphere temperature rise (*6*). The other panels show transport time corrected permafrost OC fluxes from ICDs to the 31-PC (**D**) and (uncorrected) peaks of ICD fluxes to the ESAS cores (**E**) 4-PC (*13*), PC-23 (*11*), and GC-58 (*17*). Furthermore, permafrost OC release by AL is shown for (**F**) the 31-PC and the other cores from the ESAS (**G**). The abbreviated time intervals are the DO-3, the LGM, the HS-1, the BA warm interstadial, the cold YD stadial, and the warm Holocene/Interglacial (including the EH PB). *The shown fluxes of the 31-PC include a correction for cross-shelf transport times due to increasing offshore distance with global sea-level rise during the EH (text S2). The ICD and AL fluxes indicate the minimum estimate, while pale peak shadows show the possible maximum estimate.

Dual carbon isotope–based source apportionment reveals that the BA warming by nearly 1°C in the Northern Hemisphere (Fig. 4) (*6*) triggered the remobilization of permafrost OC mainly from ICD, accounting for 64 ± 6% (*n* = 5) of the OC after 14.7 ka B.P. compared to lower and more variable amounts (38 ± 29%; *n* = 10) during DO-3, LGM, and HS-1. The flux of permafrost carbon from ICD to 31-PC more than tripled from the LGM and HS-1 baseline (0.3 ± 0.1 g m^−2^ year^−1^) to the BA (1.1 ± 0.1 g m^−2^ year^−1^). This is in line with reconstructed fluxes of ICD-OC to the Chukchi Sea (Figs. 1 and 4), which were three times higher during the late BA (13.1 to 12.9 ka B.P.) than in the Holocene (*13*). The synchronous observational records in the Chukchi Sea and on the southern Lomonosov Ridge (31-PC) together represent a wide drainage footprint (ESAS) and suggest that thawing and remobilization of OC from ICD was a large-scale phenomenon across the entire ESAS coastline and drainage basin.

Biomarker degradation proxies suggest a high degree of decomposition of the OC released by thawing ICD during the BA (fig. S2). The 3,5-Bd/V ratio (~8.1) remains in the same range of degradation during HS-1 (~8.8) and an even stronger depletion of HMW *n*-alkanoic acids relative to HMW *n*-alkanes during the BA (0.2 to 0.5) when compared with the HS-1 (0.6 and 0.7). The high degree of decomposition indicates a predominance of thaw and remobilization of OC from inland ICD on today’s ESAS by thermokarst, thermo-erosion, and, possibly, riverbank erosion (*9*), with degradation both on site and during long-distance transport to the 31-PC location. It appears that only a smaller amount of the OC was remobilized by coastal erosion resulting from global sea-level rise (~27 m) and inundation of about 10% of the total ESAS during the BA (*5*, *31*). Irrespective of the exact mobilization mechanism, the (i) highly enhanced liberation of old OC from ICD permafrost and (ii) molecular evidence for extensive degradation support the hypothesis that ICD thawing during the BA contributed to the concurrent rise of atmospheric CO_2_ and its isotopic changes (^13^C, ^14^C) (Fig. 4) (*14*, *15*). However, the exact amount of OC that was stored as ICD on exposed Arctic shelves is still challenging to estimate (*8*), and it is not yet possible to quantitatively constrain the magnitude of the fraction that got converted to CO_2_ after thaw. In summary, the BA warming at 14.7 ka B.P. activated the thawing and degradation of old Pleistocene ICD permafrost that covered the large “dry land” ESAS and thereby released large amounts of OC into active cycling.

### Massive permafrost carbon remobilization during the Early Holocene

The most striking observational evidence for a large remobilization of permafrost OC is during the EH. Isotope-traced OC and biomarker fluxes show a sudden and strong increase in deposition of released permafrost OC, arriving after long lateral transport on the Lomonosov Ridge between 10 and 7.6 ka B.P. The PF-C flux from ICD was, on average, 2.7 ± 0.6 g m^−2^ year^−1^ (10 to 9 ka B.P.; *n* = 4), which is roughly one order of magnitude higher than the flux during the LGM and HS-1 (0.3 ± 0.1 g m^−2^ year^−1^) and two to three times higher than during the BA (1.1 ± 0.1 g m^−2^ year^−1^). This is despite the fact that the 31-PC location was much further from land during the EH than during the earlier periods (text S2 and fig. S5), which likely changed the depositional dynamics and thus sedimentation rates as large parts of the ESAS were flooded during the EH. Source apportionment shows that the release of permafrost OC from active layer thawing was a minor input to sediments on the Siberian continental margin during the EH (0.7 ± 0.4 g m^−2^ year^−1^). The lignin-based source fingerprint [ratios of C/V (cinnamyl/vanillyl) and S/V] reveals that OC remobilized during the EH contained notably more angiosperm tissues (S/V, 0.53 ± 0.11; *n* = 15) than during the LGM, suggesting a more tundra-like OC source similar to organic material stored in ICD (Fig. 3) (*32*).

Our results of enhanced input of remobilized OC for the Younger Dryas (YD)–EH warming expands upon an earlier report on this (*11*). Tesi and coworkers (*11*) showed that the dominant source of the large OC input to their sampling location in the Laptev Sea was fluvially transported material from the permafrost active layer (Fig. 4). This previous study built on source apportionments in core PC-23 (Fig. 1), which is located near the mouth of the paleo-Lena river. It is likely that OC transported to this location showed a particularly strong contribution of river-transported material from inland Siberia that may not be representative for most of the ESAS. An increasing number of studies with a broader regional footprint (East Siberian Sea; Chukchi Sea) (*13*, *17*), as well as the present study, now indicate that deglacial ESAS sediments and its large OC input are dominated by remobilized ICD permafrost, which was released by EH warming and sea-level rise.

Permafrost OC remobilization during the EH caused extensive underway degradation. The OC remobilized during the EH was, however, somewhat less degraded than in the earlier periods, which is consistent with a massive pulse-like remobilization of OC. A comparison of the travel distance for terrigenous OC with modern cross-shelf degradation trends in the Laptev Sea (*33*) shows that the 3,5-Bd/V ratio in 31-PC for the EH interval between 10 and 7.6 ka B.P. (0.4 to 0.5) agrees with the 3,5-Bd/V ratio of ~0.5 at a cross-shelf transport distance of about 400 km or more (*33*). The modern system relationship between transport distance and the 3,5-Bd/V degradation status is consistent with the EH period observations as the offshore distance of the core site increased from about 200 to 400 km between 11 and 7.6 ka B.P. (*5*, *31*). However, other degradation status proxies such as the ratio of HMW *n*-alkanoic acids over HMW *n*-alkanes (0.7 ± 0.3) are even somewhat lower than expected based on the comparison above (acids/alkanes > 1.9) (*33*). Given the extent of degradation indicated by the lipid biomarkers, it seems that large part of the initially remobilized terrigenous OC during the EH was degraded during cross-shelf transport, implying release of ^14^C-depleated CO_2_ to the ocean with subsequent venting to the atmosphere.

The large global sea-level rise of ~44 m (*5*) and the inundation of the ESAS displaced the coast line by about 200 km during the EH (*31*, *34*). Considering the large offshore distance of the 31-PC location by the time of the EH, resulting in longer cross-shelf transport times, we expect a substantial time gap between permafrost remobilization and final (re-)deposition of the OC on the Lomonosov Ridge. We estimate (text S2 and fig. S5) that cross-shelf transport may delay the seaward transport of terrigenous OC by 0.6 to 2.7 ka for material released during the EH. In contrast, cross-shelf transport times during earlier periods of permafrost OC remobilization (BA and DO-3) were much shorter (below 0.2 ka; fig. S5). Accordingly, OC deposited at the 31-PC location between 10 and 7.6 ka B.P. was likely released from land in the time window 12.5 to 8.2 ka B.P., based on the two cross-shelf transport scenarios (text S2). This period thus coincides with the EH onset around 11.7 ka B.P. (Fig. 4) when northern hemispheric temperatures increased by more than 1°C (*6*), and massive permafrost OC release to Laptev Sea sediments has been demonstrated (*11*). The EH warming likely resulted in large-scale permafrost thawing and net emissions of ^14^C-depleted CO_2_ to the atmosphere.

## DISCUSSION

This new time-continuous record from the Siberian continental slope provides insights into the release of previously inactive OC from permafrost across northeastern Siberia over the last 27 ka. Characterization of OC using carbon isotopes (δ^13^C and Δ^14^C) and terrestrial biomarkers reveals that during stadial conditions (LGM, HS-1), the Siberian continental margin received low inputs of fluvially transported terrigenous OC. By contrast, three pulses of permafrost OC release coincide with the three last largest warming events recorded in Greenland ice cores (*26*). Abrupt climate warming during the DO-3 event appears to have caused a large remobilization of relatively young OC from thawing of predominantly permafrost active layer with subsequent fluvial transport to the Arctic Ocean. Warming and global sea-level rise during the deglaciation (BA and EH) caused remobilization of much older OC from ICD permafrost on the broad aerially exposed Siberian shelf, via thermokarst formation, riverbank erosion, and coastal thermo-erosion. This occurred in two stages: (i) during the BA (14.7 to 12.9 ka B.P.) where reconstructed fluxes of terrigenous biomarkers and OC source fractions reveal permafrost OC release two to three times higher than during the LGM and (ii) an even larger release during the EH (centered around ~11.7 ka B.P.), with fluxes one order of magnitude higher than during stadial conditions (LGM, HS-1). Biomarker degradation proxies suggest strong underway OC degradation throughout the 31-PC, which implies that massive OC release during these three warming pulses (DO-3, BA, and EH) not only transferred large amounts of old OC from terrestrial to aquatic systems but also caused enhanced OC degradation with resulting emissions of ^14^C-depleated CO_2_ to the atmosphere.

The results from this study on large-scale OC remobilization from permafrost are consistent with a growing set of observational records from the Arctic Ocean and provide support for modeling studies that simulated large injections of CO_2_ into the atmosphere during deglaciation (*14*–*16*). This demonstrates that Arctic warming by only a few degrees may suffice to abruptly activate large-scale permafrost thawing, indicating a sensitive trigger for a threshold-like permafrost climate change feedback.

## MATERIALS AND METHODS

### Study area and large-scale carbon sources

The Siberian continental margin is the northern margin of the Eurasian continent and recipient of large terrestrial export of sediments and OC (Fig. 1). Taken together, the shelf area of the Laptev, East Siberian, and Russian Chukchi Seas constitutes the world’s largest continental shelf area, i.e., the ESAS, where about half of the area is shallower than 50 m depth (*31*). This region today receives terrigenous OC both from erosion of permafrost coastlines and from riverine transport of permafrost material from continental Siberia (*20*), of which OC from thermo-erosion of ICD exceeds OC released from the permafrost active layer and riverine transport by a factor of 2 (*20*, *35*). Within the boundaries of the shelf, terrigenous OC input strongly influences the ESAS water chemistry (*36*) and dominates the composition of ESAS surface sediment OC (*32*, *33*). Beyond the shelf edge, marine planktonic OC sources become a larger contributor to the sediment OC pool (*33*).

### Sampling

The sediment core SWERUS-L2-31-PC1 was collected on 2014-09-15 in 1120 m water depth (79°54.89′N, 143°14.01′E) during the Swedish-Russian-U.S. Investigation of Climate, Cryosphere, and Carbon interactions in the East Siberian Arctic Ocean (SWERUS-C3) program onboard the Swedish icebreaker *Oden*. Basic physical (bulk density and magnetic susceptibility) and geochemical analyses (x-ray fluorescence spectroscopy) were performed shipboard using a Multi-Sensor Core Logger (Geotek, UK) and onshore using an ITRAX core scanner (Cox Analytical, Sweden), respectively. Subsampling for OC characterization (OC, δ^13^C, Δ^14^C, and molecular biomarkers) at 10-cm resolution was carried out at Stockholm University.

### Age-depth model of core 31-PC

The sedimentation history of the 8-m-long core 31-PC encompasses the last 27 ka (fig. S1) and builds on stratigraphic correlation to Greenland ice-core records within the constraints of radiocarbon dating, using a novel Bayesian probabilistic alignment method (*19*). Accordingly, this environmental record starts at the onset of early Marine Isotope Stage (MIS-2), which includes the entire LGM (26.5 to 20 ka B.P.) (*5*), the early deglaciation including the HS-1 (late MIS-2; 20 to 14.7 ka B.P.), the BA warm interval (late MIS-2; 14.7 to 12.9 ka B.P.), the YD cold interval (late MIS-2; 12.9 to 11.7 ka B.P.), the rapid warming transition into the EH, and then the entire Holocene (MIS-1; 11.7 ka B.P. to today). Details about the age model construction are presented in a previous study (*19*).

### Carbon isotope analysis

Stable carbon isotope (δ^13^C) ratios are a powerful tool to distinguish between terrigenous and marine sources of OC. Ratios around −27‰ typically characterize terrigenous biomass in marine sediment OC, whereas phytoplankton usually distributes around −21‰. For the current study, we analyzed 80 freeze-dried and homogenized bulk sediment samples for δ^13^C and OC. For this purpose, ~10 mg of sediment was weighed in silver capsules and acidified with 3 M HCl to remove carbonates. Analyses were performed using a Carlo Erba NC2500 elemental analyzer coupled to an isotope-ratio mass spectrometer (Finnigan DeltaV Advantage) in the Department of Geological Sciences, Stockholm University. The OC concentrations were previously published in a paper describing the 31-PC chronology (*19*).

Radiocarbon ages (^14^C) of OC provide additional information about the source contributions of different terrestrial OC pools given the large age offset between more contemporary permafrost soils and strongly pre-aged permafrost deposits from the Late Pleistocene. A subset of 27 samples was sent to the U.S. National Ocean Science Accelerator Mass Spectrometry (NOSAMS) facility at Woods Hole Oceanographic Institution (Woods Hole, MA, USA) for ^14^C analysis. To remove carbonates, the sediments were treated with HCl vapor at NOSAMS. To calculate the predepositional ^14^C activity of the organic matter, we accounted for the ^14^C decay of the OC after the year of deposition (Yd) provided by the age-depth model (*19*), following Eq. 1

$$\Delta^{14}C=(\text{Fm}\times e^{\lambda \;(1950-\text{Yd})}-1)\times 1000$$ (1)

where Fm is the fraction modern provided by the AMS and λ is the decay constant, i.e., 1/(true mean life) of radiocarbon (1/8267). Accordingly, the predepositional ages represent the age gap between fixation of the atmospheric ^14^C-CO_2_ and deposition at the core location.

### Specific mineral surface area of the sediment samples

For sediment surface area (SA) analysis, an aliquot of about 0.7 g of freeze-dried sediment was heated to 400°C for 12 hours to combust all organic matter. After cooling for at least 10 hours, the samples were repeatedly washed with 50 ml of Milli-Q water and pelletized in a centrifuge for 10 min at 8000 rpm for removal of water, followed by 48 hours of freeze drying for complete water removal. Before SA analysis, the samples were degassed under N_2_ purge at 200°C for at least 2 hours. Specific SA was finally measured by six pressure point measurements on a Micromeritics Gemini VII SA and Porosity analyzer. The instrument performance was monitored regularly using two reference materials (black carbon and TiO_2_) provided by Micromeritics.

### Molecular biomarker analysis: CuO products and lignin phenols

We used lignin phenols as one of several means to trace terrigenous OC in marine sediments (*24*). A CuO oxidation protocol (*32*) using a microwave system was used for biopolymer breakup. Depending on the total OC content, 1 to 1.8 g of freeze-dried sediment were loaded into Teflon tubes, mixed with 300 mg of cupric oxide (CuO) and 50 mg of ammonium iron (II) sulfate hexahydrate [(NH_4_)_2_Fe(SO_4_)_2_·6 H_2_O], and suspended in N_2_-purged 2 M NaOH solution. The extraction program used an UltraWAVE Milestone 215 microwave digestion system using a program of 90-min duration at 130°C. After extraction, an internal recovery standard (ethyl-vanillin, cinnamic acid) was added and samples were acidified to pH 1 using concentrated HCl. Afterward, phenols were extracted from the water phase using ethyl acetate (EtOAc) and residual water was removed from the supernatant using anhydrous sodium sulfate (Na_2_SO_4_). The EtOAc was evaporated in a CentriVap (Christ RVC 2-25) at 60°C for 1 hour and redissolved in pyridine.

For analysis, the extracts were derivatized using bis-trimethylsilyl trifluoroacetoamide (BSTFA) and 1% trimethylchlorosilane (TMCS) to silylate exchangeable hydrogen atoms and then analyzed in single ion monitoring (SIM) mode on a gas chromatograph (GC) with mass spectrometer (GC-MS) detector (GC-MS 7820A, Agilent Technologies USA) using a DB1-MS column (30 m × 250 μm; 0.25 μm film thickness). The GC program had an initial temperature of 50°C and a subsequent heating ramp of 10°C per minute until 300°C. Individual compound concentrations were calculated using a six-point calibration of known concentrations of commercially available external standards. We report total lignin concentrations as a sum of vanillin (Vl), acetovanillone (Vn), vanillic acid (Vd), syringaldehyde (Sl), acetosyringone (Sn), syringic acid (Sd), *p*-coumaric acid (pCd), and ferulic acid (Fd) normalized to SA (ng m^−2^) and flux rates per square meter sea floor area (mg m^−2^ year^−1^) in table S2. In addition, we used the compound 3,5-dihydrobenzoic acid (3,5-Bd) to assess the degradation and removal of vanillyl lignin phenols (V) relative to 3,5-Bd.

### Molecular biomarker analysis: Solvent-extractable lipids

Vascular plant lipid biomarkers (HMW *n*-alkanes and *n*-alkanoic acids) were also analyzed at 21 depths to characterize the input and degradation status of terrigenous organic matter. Both compound classes occur primarily in terrigenous organic matter and provide a number of proxies that provide detail about OC degradation. The extraction of lipid biomarkers included accelerated solvent extraction (ASE; Dionex ASE 300) of sediment samples and a clean-up protocol following previously described procedures (*37*). Briefly, an aliquot of about 3 g of freeze-dried sediment was packed in ASE steel cells with precombusted glass fiber filters and glass wool and amended with internal recovery standards (*d*_50_-tetracosane, 2-hexadecanol, and *d*_39_-eicosanoic acid). The ASE was performed using three extraction cycles with a mixture of dichloromethane:methanol (9:1, v/v) at 85°C for 5 min at 100 bar with a flush volume of 60% and a purge time of 60 s.

The total solvent extracts were then treated with acid-activated Cu and pre-combusted Na_2_SO_4_ (anhydrous) and allowed to react at room temperature overnight for sulfur and water removal, respectively. After solvent reduction using a rotary evaporator, the concentrated extracts were separated into neutral and acid fractions over BondElut cartridges (Agilent Technologies USA) using 15 ml of dichloromethane:isopropanol (2:1) and 15 ml of diethyl ether with 2% acetic acid, respectively. Using an additional column packed with 100% activated Al_2_O_3_ (0.5 g of 100% activated Al_2_O_3_; grain size of 0.063 to 0.2 mm; activation by baking at 420°C), the neutral fraction was further fractionated with 3 ml of the eluents hexane:dichloromethane (9:1), resulting in a nonpolar fraction (including *n*-alkanes), and 3 ml of methanol:dichloromethane (1:1) to elute a fraction of polar compounds. All resulting fractions were reduced under N_2_ stream and spiked with a known amount of injection standard (*p*-terphenyl) to calculate the final volume before analysis. The acid fraction was derivatized using BSTFA + 1% TMCS before analysis using GC-MS.

The nonpolar (*n*-alkanes) and acid fractions (*n*-alkanoic acids) were analyzed on a GC-MS (GC-MS 7820A, Agilent Technologies USA) in splitless mode using a DB-5 column (30 m, 0.25-μm film, 0.3 mm) and a temperature program starting at 60°C with a temperature gradient of 10°C per minute until 310°C, which was kept for 16 min. Integrated peak signals from SIM were quantified using seven-point calibration of external quantification standards of long-chained *n*-alkanes and *n*-alkanoic acids. The recoveries of the internal standards were 92 ± 12% for the nonpolar fractions and 57 ± 20% for the acid fractions. As for lignin phenols, this study reports concentrations of HMW *n*-alkanes (ΣC_23_-C_33_) and *n*-alkanoic acids (ΣC_24_-C_32_) as normalized to SA (ng m^−2^) and as flux rates (mg m^−2^ year^−1^). We also report the carbon preference index (CPI; table S3) as a degradation proxy of terrigenous OC for both compound classes [CPI_alk_ for HMW *n*-alkanes; CPI_acid_ for HMW *n*-alkanoic acids; (*33*)], which is based on the degree of the odd-over-even predominance of HMW *n*-alkane homologues and the even-over-odd predominance of HMW *n*-alkanoic acids. Both decrease toward 1 with degradation.

### Statistical method for source apportionment of OC

This study uses a Bayesian statistical approach (*21*) to calculate the relative fractions derived from the major OC sources (i.e., ICD, permafrost active layer, and marine biomass) using the δ^13^C-OC and Δ^14^C-OC values in core 31-PC (fig. S3). This method applies a dual-isotope mass balance with three end members in a Markov chain Monte Carlo simulation (*21*) using Matlab R2018a with 1,000,000 runs and a burn-in period of 10,000 runs per sample. The isotopic definition of the end members was based on an extensive literature survey of δ^13^C and Δ^14^C values measured in potential OC sources following the approach used in a number of previous ESAS studies (*11*, *13*, *20*). Each end member consists of an isotopic mean ± SD for both Δ^14^C-OC and δ^13^C-OC and thus accounts for the natural end member variability. Analytical errors are not included in the mixing model, as these are considered negligible compared to the uncertainties of the end member variability. For marine biomass, Δ^14^C-OC of −50 ± 12‰ (*n* = 5) and δ^13^C-OC of −21.0 ± 2.6‰ (*n* = 31) was used, representing an open-marine Arctic environment (*13*). The permafrost active layer end member was based on active layer observations from Siberia (maximum of 1 m depth), with Δ^14^C-OC of −197.5 ± 148.3‰ (*n* = 60; mean ± SD) and δ^13^C-OC of −26.4 ± 0.8‰ (*n* = 56; fig. S3) (*22*). The δ^13^C-OC of the ICD end member was constrained on the basis of a previous review on ICD exposures in Siberia (−26.3 ± 0.7‰; *n* = 374) (*23*). For the Δ^14^C-OC of ICD, however, we needed to consider the temporal depth of the 31-PC record (i.e., 27 ka B.P.), which overlaps with the period of active ICD formation ca. 120 to 10 ka B.P. (*23*, *38*), as well as the limits of radiocarbon dating that allow no consideration of deposits older than ca. 50 ka. We therefore used a previously published database of Δ^14^C-OC in Siberian ICD exposures (−962 ± 61‰; *n* = 415) (*22*) to assess the variability in ICD formation over time. On the basis of this assessment, this study describes the ICD end member assuming a uniform distribution of ICD ^14^C ages that formed between the last interglacial and the Holocene (120 to10 ka B.P.; see text S2 for details). This study also accounts for the aging of terrigenous OC due to cross-shelf transport as a result of global sea-level changes and changing distance between the 31-PC location and the coast line. While this affects the Δ^14^C-OC of the active layer and ICD end members, the δ^13^C-OC values for both terrigenous OC end members were kept constant throughout the record. A full description of the ICD end member design is provided in text S1, and more details about the calculations of cross-shelf transport times are provided in text S2. All resulting source fractions are presented in fig. S3 and table S4.

## Supplementary Material

abb6546_SM.pdf

## SUPPLEMENTARY MATERIALS

Supplementary material for this article is available at http://advances.sciencemag.org/cgi/content/full/6/42/eabb6546/DC1

## References

[1] IPCC, *IPCC Special Report on Ocean and Cryosphere: Chapter 3: Polar Regions* (IPCC, 2019).

[2] Hugelius G., Strauss J., Zubrzycki S., Harden J. W., Schuur E. A. G., Ping C.-L., Schirrmeister L., Grosse G., Michaelson G. J., Koven C. D., O’Donnell J. A., Elberling B., Mishra U., Camill P., Yu Z., Palmtag J., Kuhry P., Estimated stocks of circumpolar permafrost carbon with quantified uncertainty ranges and identified data gaps. Biogeosciences 11, 6573–6593 (2014).

[3] Vonk J. E., Gustafsson Ö., Permafrost-carbon complexities. Nat. Geosci. 6, 675–676 (2013).

[4] Turetsky M. R., Abbott B. W., Jones M. C., Anthony K. W., Olefeldt D., Schuur E. A. G., Grosse G., Kuhry P., Hugelius G., Koven C., Lawrence D. M., Gibson C., Sannel A. B. K., McGuire A. D., Carbon release through abrupt permafrost thaw. Nat. Geosci. 13, 138–143 (2020).

[5] Lambeck K., Rouby H., Purcell A., Sun Y., Sambridge M., Sea level and global ice volumes from the Last Glacial Maximum to the Holocene. Proc. Natl. Acad. Sci. U.S.A. 111, 15296–15303 (2014). 25313072 10.1073/pnas.1411762111 PMC4217469

[6] Shakun J. D., Clark P. U., He F., Marcott S. A., Mix A. C., Liu Z., Otto-Bliesner B., Schmittner A., Bard E., Global warming preceded by increasing carbon dioxide concentrations during the last deglaciation. Nature 484, 49–54 (2012). 22481357 10.1038/nature10915

[7] Schmitt J., Schneider R., Elsig J., Leuenberger D., Lourantou A., Chappellaz J., Köhler P., Joos F., Stocker T. F., Leuenberger M., Fischer H., Carbon isotope constraints on the deglacial CO_2_ rise from ice cores. Science 336, 711–714 (2012). 22461496 10.1126/science.1217161

[8] Lindgren A., Hugelius G., Kuhry P., Extensive loss of past permafrost carbon but a net accumulation into present-day soils. Nature 560, 219–222 (2018). 30069043 10.1038/s41586-018-0371-0

[9] Romanovskii N. N., Hubberten H.-W., Gavrilov A. V., Tumskoy V. E., Tipenko G. S., Grigoriev M. N., Siegert C., Thermokarst and land-ocean interactions, Laptev sea region, Russia. Permafr. Periglac. Process. 11, 137–152 (2000).

[10] Strauss J., Schirrmeister L., Grosse G., Fortier D., Hugelius G., Knoblauch C., Romanovsky V., Schädel C., von Deimling T. S., Schuur E. A. G., Shmelev D., Ulrich M., Veremeeva A., Deep Yedoma permafrost: A synthesis of depositional characteristics and carbon vulnerability. Earth Sci. Rev. 172, 75–86 (2017).

[11] Tesi T., Muschitiello F., Smittenberg R. H., Jakobsson M., Vonk J. E., Hill P., Andersson A., Kirchner N., Noormets R., Dudarev O., Semiletov I., Gustafsson Ö., Massive remobilization of permafrost carbon during post-glacial warming. Nat. Commun. 7, 13653 (2016). 27897191 10.1038/ncomms13653 PMC5141343

[12] Gaglioti B. V., Mann D. H., Jones B. M., Pohlman J. W., Kunz M. L., Wooller M. J., Radiocarbon age-offsets in an arctic lake reveal the long-term response of permafrost carbon to climate change. J. Geophys. Res. Biogeosci. 119, 1630–1651 (2014).

[13] Martens J., Wild B., Pearce C., Tesi T., Andersson A., Bröder L., O’Regan M., Jakobsson M., Sköld M., Gemery L., Cronin T. M., Semiletov I., Dudarev O. V., Gustafsson Ö., Remobilization of old permafrost carbon to Chukchi Sea sediments during the end of the last deglaciation. Global Biogeochem. Cycles 33, 2–14 (2019). 31007381 10.1029/2018GB005969 PMC6472570

[14] Winterfeld M., Mollenhauer G., Dummann W., Köhler P., Lembke-Jene L., Meyer V. D., Hefter J., McIntyre C., Wacker L., Kokfelt U., Tiedemann R., Deglacial mobilization of pre-aged terrestrial carbon from degrading permafrost. Nat. Commun. 9, 3666 (2018). 30201999 10.1038/s41467-018-06080-w PMC6131488

[15] Köhler P., Knorr G., Bard E., Permafrost thawing as a possible source of abrupt carbon release at the onset of the Bølling/Allerød. Nat. Commun. 5, 5520 (2014). 25409739 10.1038/ncomms6520 PMC4263146

[16] Crichton K. A., Bouttes N., Roche D. M., Chappellaz J., Krinner G., Permafrost carbon as a missing link to explain CO_2_ changes during the last deglaciation. Nat. Geosci. 9, 683–686 (2016).

[17] Keskitalo K., Tesi T., Bröder L., Andersson A., Pearce C., Sköld M., Semiletov I. P., Dudarev O. V., Gustafsson Ö., Sources and characteristics of terrestrial carbon in Holocene-scale sediments of the East Siberian Sea. Clim. Past 13, 1213–1226 (2017).

[18] Meyer V. D., Hefter J., Köhler P., Tiedemann R., Gersonde R., Wacker L., Mollenhauer G., Permafrost-carbon mobilization in Beringia caused by deglacial meltwater runoff, sea-level rise and warming. Environ. Res. Lett. 14, 085003 (2019).

[19] Muschitiello F., O’Regan M., Martens J., West G., Gustafsson Ö., Jakobsson M., A new 30 000-year chronology for rapidly deposited sediments on the Lomonosov Ridge using bulk radiocarbon dating and probabilistic stratigraphic alignment. Geochronology 2, 81–91 (2020).

[20] Vonk J. E., Sánchez-García L., van Dongen B. E., Alling V., Kosmach D., Charkin A., Semiletov I. P., Dudarev O. V., Shakhova N., Roos P., Eglinton T. I., Andersson A., Gustafsson Ö., Activation of old carbon by erosion of coastal and subsea permafrost in Arctic Siberia. Nature 489, 137–140 (2012). 22932271 10.1038/nature11392

[21] Andersson A., Deng J., Du K., Zheng M., Yan C., Sköld M., Gustafsson Ö., Regionally-varying combustion sources of the january 2013 severe haze events over eastern China. Environ. Sci. Technol. 49, 2038–2043 (2015). 25569822 10.1021/es503855e

[22] Wild B., Andersson A., Bröder L., Vonk J., Hugelius G., McClelland J. W., Song W., Raymond P. A., Gustafsson Ö., Rivers across the Siberian Arctic unearth the patterns of carbon release from thawing permafrost. Proc. Natl. Acad. Sci. U.S.A. 116, 10280–10285 (2019). 31061130 10.1073/pnas.1811797116 PMC6535028

[23] Schirrmeister L., Kunitsky V., Grosse G., Wetterich S., Meyer H., Schwamborn G., Babiy O., Derevyagin A., Siegert C., Sedimentary characteristics and origin of the Late Pleistocene Ice Complex on north-east Siberian Arctic coastal lowlands and islands - A review. Quat. Int. 241, 3–25 (2011).

[24] Goñi M. A., Hedges J. I., Sources and reactivities of marine-derived organic matter in coastal sediments as determined by alkaline CuO oxidation. Geochim. Cosmochim. Acta 59, 2965–2981 (1995).

[25] Winterfeld M., Goñi M. A., Just J., Hefter J., Mollenhauer G., Characterization of particulate organic matter in the Lena River delta and adjacent nearshore zone, NE Siberia–Part 2: Lignin-derived phenol compositions. Biogeosciences 12, 2261–2283 (2015).

[26] Dansgaard W., Johnsen S. J., Clausen H. B., Dahl-Jensen D., Gundestrup N. S., Hammer C. U., Hvidberg C. S., Steffensen J. P., Sveinbjörnsdottir A. E., Jouzel J., Bond G., Evidence for general instability of past climate from a 250-kyr ice-core record. Nature 364, 218–220 (1993).

[27] Prokopenko A. A., Williams D. F., Karabanov E. B., Khursevich G. K., Continental response to Heinrich events and Bond cycles in sedimentary record of Lake Baikal, Siberia. Glob. Planet. Change 28, 217–226 (2001).

[28] Andreev A. A., Schirrmeister L., Tarasov P. E., Ganopolski A., Brovkin V., Siegert C., Wetterich S., Hubberten H.-W., Vegetation and climate history in the Laptev Sea region (Arctic Siberia) during Late Quaternary inferred from pollen records. Quat. Sci. Rev. 30, 2182–2199 (2011).

[29] Stein R., Boucsein B., Fahl K., Garcia de Oteyza T., Knies J., Niessen F., Accumulation of particulate organic carbon at the Eurasian continental margin during late Quaternary times: Controlling mechanisms and paleoenvironmental significance. Glob. Planet. Change 31, 87–104 (2001).

[30] Jakobsson M., Andreassen K., Bjarnadóttir L. R., Dove D., Dowdeswell J. A., England J. H., Funder S., Hogan K., Ingólfsson Ó., Jennings A., Larsen N. K., Kirchner N., Landvik J. Y., Mayer L., Mikkelsen N., Möller P., Niessen F., Nilsson J., O’Regan M., Polyak L., Nørgaard-Pedersen N., Stein R., Arctic Ocean glacial history. Quat. Sci. Rev. 92, 40–67 (2014).

[31] Jakobsson M., Mayer L., Coakley B., Dowdeswell J. A., Forbes S., Fridman B., Hodnesdal H., Noormets R., Pedersen R., Rebesco M., Schenke H. W., Zarayskaya Y., Accettella D., Armstrong A., Anderson R. M., Bienhoff P., Camerlenghi A., Church I., Edwards M., Gardner J. V., Hall J. K., Hell B., Hestvik O., Kristoffersen Y., Marcussen C., Mohammad R., Mosher D., Nghiem S. V., Pedrosa M. T., Travaglini P. G., Weatherall P., The International Bathymetric Chart of the Arctic Ocean (IBCAO) Version 3.0. Geophys. Res. Lett. 39, L12609 (2012).

[32] Tesi T., Semiletov I., Hugelius G., Dudarev O., Kuhry P., Gustafsson Ö., Composition and fate of terrigenous organic matter along the Arctic land–ocean continuum in East Siberia: Insights from biomarkers and carbon isotopes. Geochim. Cosmochim. Acta 133, 235–256 (2014).

[33] Bröder L., Tesi T., Salvadó J. A., Semiletov I. P., Dudarev O. V., Gustafsson Ö., Fate of terrigenous organic matter across the Laptev Sea from the mouth of the Lena River to the deep sea of the Arctic interior. Biogeosciences 13, 5003–5019 (2016).

[34] Bauch H. A., Mueller-Lupp T., Taldenkova E., Spielhagen R. F., Kassens H., Grootes P. M., Thiede J., Heinemeier J., Petryashov V. V., Chronology of the Holocene transgression at the North Siberian margin. Glob. Planet. Change 31, 125–139 (2001).

[35] Semiletov I. P., Pipko I. I., Shakhova N. E., Dudarev O. V., Pugach S. P., Charkin A. N., McRoy C. P., Kosmach D., Gustafsson Ö., Carbon transport by the Lena River from its headwaters to the Arctic Ocean, with emphasis on fluvial input of terrestrial particulate organic carbon vs. carbon transport by coastal erosion. Biogeosciences 8, 2407–2426 (2011).

[36] Semiletov I., Dudarev O., Luchin V., Charkin A., Shin K.-H., Tanaka N., The East Siberian Sea as a transition zone between Pacific-derived waters and Arctic shelf waters. Geophys. Res. Lett. 32, L10614 (2005).

[37] van Dongen B. E., Semiletov I., Weijers J. W. H., Gustafsson Ö., Contrasting lipid biomarker composition of terrestrial organic matter exported from across the Eurasian Arctic by the five great Russian Arctic rivers. Global Biogeochem. Cycles 22, GB1011 (2008).

[38] Wetterich S., Tumskoy V., Rudaya N., Kuznetsov V., Maksimov F., Opel T., Meyer H., Andreev A. A., Schirrmeister L., Ice Complex permafrost of MIS5 age in the Dmitry Laptev Strait coastal region (East Siberian Arctic). Quat. Sci. Rev. 147, 298–311 (2016).

[39] North Greenland Ice Core Project members, High-resolution record of Northern Hemisphere climate extending into the last interglacial period. Nature 431, 147–151 (2004). 15356621 10.1038/nature02805

[40] Bröder L., Tesi T., Andersson A., Semiletov I., Gustafsson Ö., Bounding cross-shelf transport time and degradation in Siberian-Arctic land-ocean carbon transfer. Nat. Commun. 9, 806 (2018). 29476050 10.1038/s41467-018-03192-1 PMC5824890

