# Supplementary material for: Remobilization of dormant carbon from Siberian-Arctic permafrost during three past warming events
Source: Sci Adv. 2020 Oct 16;6(42):eabb6546. doi: 10.1126/sciadv.abb6546 (PMC7567595; doi:10.1126/sciadv.abb6546)
Supplement: abb6546_SM.pdf [file abb6546_SM.pdf]

Science Advances
AAAS

advances.sciencemag.org/cgi/content/full/6/42/eabb6546/DC1

Supplementary Materials for

Remobilization of dormant carbon from Siberian-Arctic permafrost during three past warming events

Jannik Martens*, Birgit Wild, Francesco Muschitiello, Matt O'Regan, Martin Jakobsson, Igor Semiletov, Oleg V. Dudarev, Örjan Gustafsson*

*Corresponding author. Email: orjan.gustafsson@aces.su.se (Ö.G.); jannik.martens@aces.su.se (J.M.)

Published 16 October 2020, *Sci. Adv.* **6**, eabb6546 (2020)
DOI: 10.1126/sciadv.abb6546

This PDF file includes:

Texts S1 and S2
Figs. S1 to S5
Tables S1 to S5

Text S1: Radiocarbon end member constraints for carbon from Ice Complex Deposit Permafrost

The period of ICD formation extended from the late Pleistocene to the early Holocene (10 kyr BP), and consequently overlaps with the deposition of the deeper part of the 31-PC. Accordingly, we need to consider the changing age of the ICD end member relative to the model age of the 31-PC samples. Another complication is that the detection limit of radiocarbon (~50 kyr BP) allows no consideration of deposits that accumulated >50 kyr BP, while previous research using ^{230}Th/U dating has shown that the onset of ICD formation was much earlier, namely after the last interglacial around 126-89 kyr BP (*38*). However, the dataset on Δ^{14}C values in Siberian ICD used previously for source apportionment (*20*, *22*) suggests a fairly uniform formation of ICD between the early Holocene (10 kyr BP) and the ^{14}C dating limit of ~50 kyr BP. We therefore applied a uniformly distributed function (Eq. 2) to determine the mean ^{14}C activity of ICD that was formed between a starting point *start* and its termination *end*. The *end* point is provided by the minimum age of the ICD data base (i.e. 10 kyr BP) while the onset (*start)* of ICD formation was after the last interglacial (Eemian) around 120 kyr BP. The onset age builds on published research that presents ^{230}Th/U ages of intact ICD of 126-89 kyr BP (*38*), which was at a time when the climate turned from warm interglacial to cold stadial conditions (early Weichselian; 100-50 kyr BP) (*28*). Because the last interglacial was warmer than today (*28*) we disregard any deposits older than the last interglacial.

The distribution function for the ICD end member is described as follows:

$$\mu = \int_{\exp(-\lambda\cdot start)}^{\exp(-\lambda\cdot end)} \frac{dy}{\lambda\cdot(start-end)} = \frac{\exp(-\lambda\cdot end)-\exp(-\lambda\cdot start)}{\lambda\cdot(start-end)} \quad \text{(Eq. 2)}$$

Where μ is the mean ^{14}C activity of the probability density function as *Fm* and λ is the decay constant, i.e. 1/(true mean-life) of radiocarbon (1/8267). We evaluated this equation for the part of our ICD end member database that represents finite ^{14}C ages, which lie between 11.2

to 58.4 kyr BP. It can be shown that applying Eq. 2 with 58.4 kyr as onset (*start*) and termination (*end*) 11.2 kyr results in the same $\Delta^{14}C$ of -950‰ as the simple mean of all finite $\Delta^{14}C$ data base entries (n = 317).

To account for the changing age of the ICD end member relative to the 31-PC model age, we subtracted the model age from the onset (*start*) and closing (*end*) of the ICD formation. For 31-PC samples older than 10 kyr BP the starting point was equal to 0. The uncertainty of the ICD end member was kept constant at 60‰, calculated based on the original data base on ICD $\Delta^{14}C$ values.

Text S2: Estimation of cross-shelf transport times

Previous studies have stressed that cross-shelf transport causes aging of terrigenous OC (*40*). The $\Delta^{14}C$ values of the two terrigenous end members ICD and active layer were consequently varied over time depending on the increasing cross-shelf distance offshore of the 31-PC location, using the factor of 5.5 yr/km which was determined in a previous study for a 600 km long Laptev Sea transect by correlation of transport distance and ^{14}C-dated terrigenous biomarkers (*40*). The cross-shelf distance for the 31-PC location was reconstructed for each time step using data on historical sea-level rise (*5*) and the bathymetry of the Arctic Ocean (*31*). To correct for this aging during cross-shelf transport we used a minimum estimate for the distance between the New Siberian Islands and the shelf edge next to the 31-PC location as shown in Fig. S5 for the time steps at 27, 14.7, 11.7 kyr BP and modern conditions. The resulting distance, which varied between 25 km at the LGM and 340 km at the present day, was translated into cross-shelf transport time (τ) and resulted in a modification of the $\Delta^{14}C$-ICD end member from -980 ± 60‰ at the present day to -912 ± 60‰ at 27 kyr BP (Fig. S3). For the active layer $\Delta^{14}C$, the endmember changed from -360 ± 118‰ at the present day to -208 ± 146‰ at 27 kyr BP.

Finally, the mean (μ) and standard deviation (*σ)* of the ICD and active layer end members can be calculated using the equation below, from the previous assessment of cross-shelf transport times (*40*):

$$\mu_\tau = \mu_0 \times e^{-\tau/8267} \text{ (Eq. 3)}$$

$$\sigma_\tau = \sigma_0 \times e^{-\tau/8267} \text{ (Eq. 4)}$$

Cross-shelf transport is causes peak delay of terrigenous OC to the 31-PC. To estimate the minimum and maximum delay, we assume two contrasting routes for cross-shelf transport (Fig. S5), i) a straight line from the New Siberian Islands to the shelf edge pointing to the 31-PC location, and ii) a line from the Lena delta northwards to the paleo-river mouth, and then onwards and parallel to the paleo-coastline to the same shelf edge point. Because the Lena and Indigirka rivers drained near-directly into the Central Arctic Ocean at 14.7 and 27 kyr BP, and (paleo-)topographic barriers prevented any coast-parallel transport on the paleo-shelf, we assume no significant delay of terrigenous OC transported by Arctic rivers at these times and used the cross-shelf transect from the New Siberian Islands as a maximum constraint. The resulting cross-shelf transport times (CSTT) for each of the periods of PF-C remobilization are summarized below:

Remobilization event	Min. distance (km)	Min. CSTT (kyr)	Max. distance (km)	Max. CSTT (kyr)
11.7 kyr BP	104.7	0.57	481	2.65
14.7 kyr BP	-	-	34.7	0.19
27 kyr BP	-	-	27.8	0.15

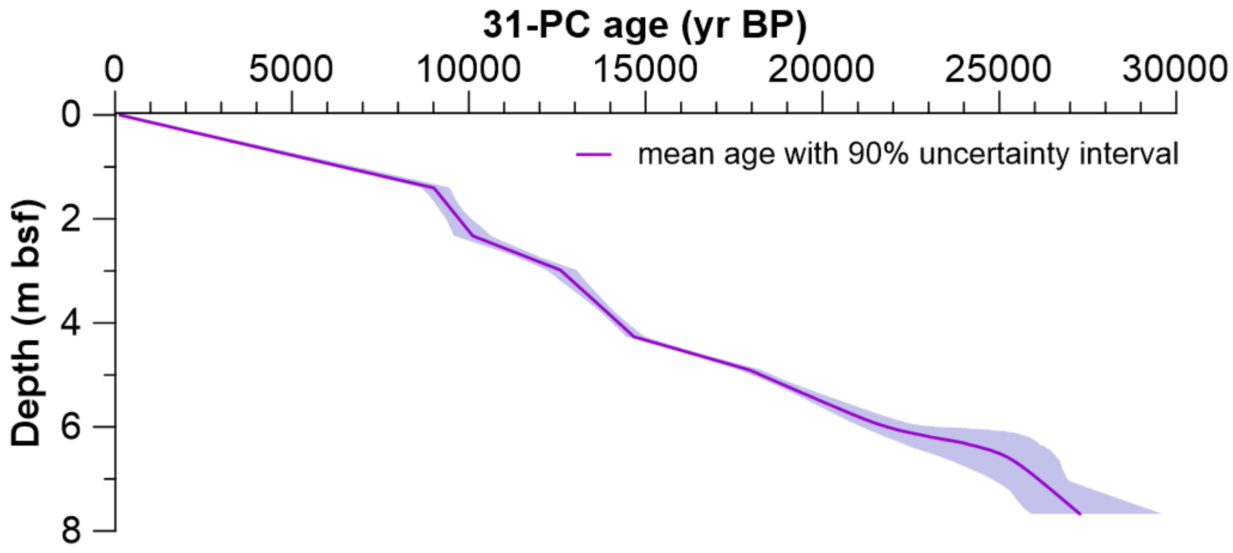

Fig. S1: Plot of the down-core chronology of the 31-PC from a previous study (*19*). Shown are the median ages and the 90% uncertainty envelope of the age model.

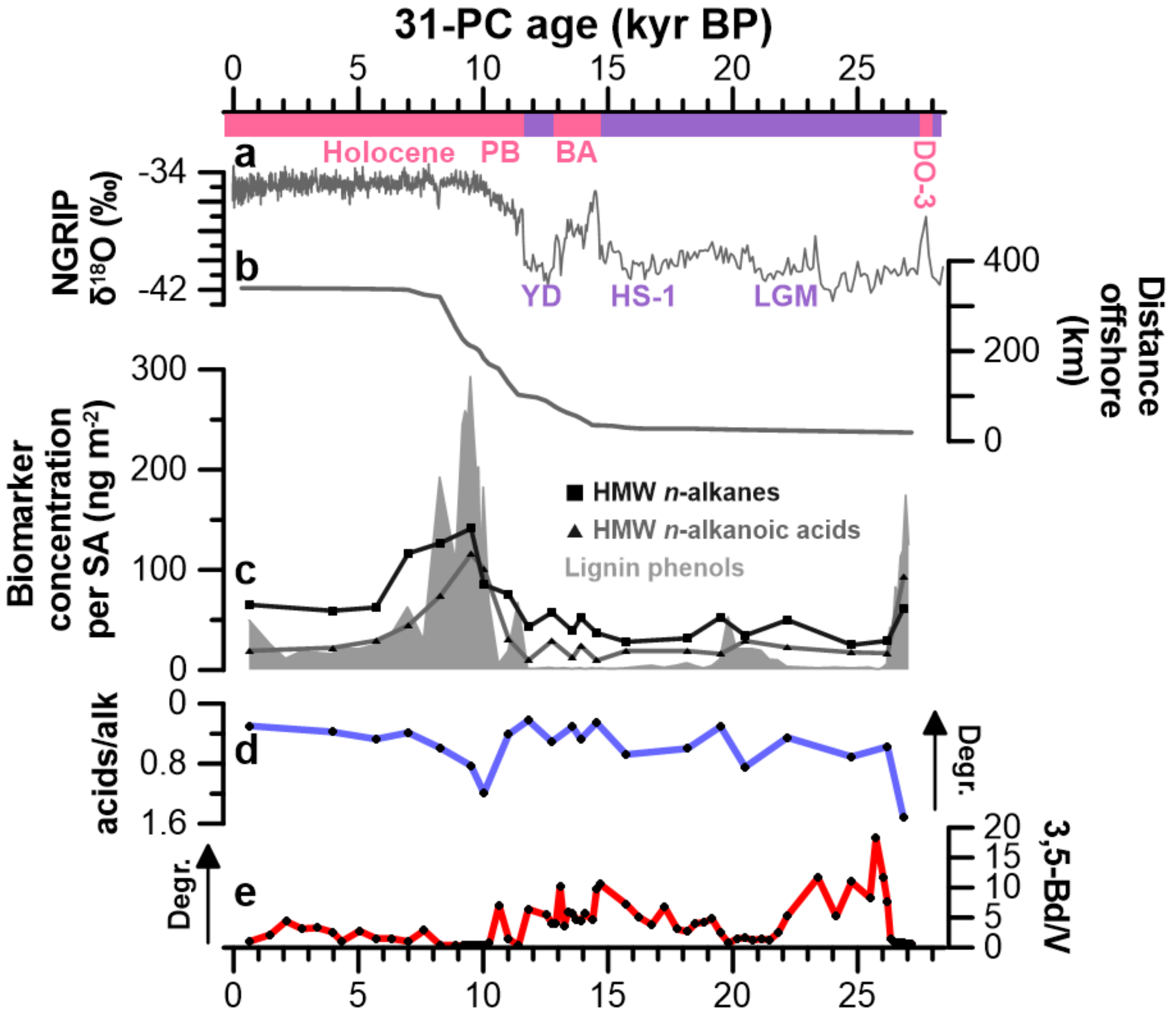

Fig. S2: Concentrations and degradation proxies based on lignin phenols and high molecular weight (HMW) *n*-alkanes and *n*-alkanoic acids. The biomarker proxies are compared with (a) the Greenland ice core $\delta^{18}O$ record (NGRIP, 2004) and (b) the offshore distance of the 31-PC location. For the 31-PC (c) the concentrations to surface area (SA) of lignin, HMW *n*-alkanes and HMW *n*-alkanoic acids are shown, as well as (d) the relative abundance of HMW *n*-alkanoic acids over HMW *n*-alkanes (acids/alk) and (e) the 3,5-Bd over vanillyl degradation proxy. Arrows indicate how increasing degradation affects the proxies. This figure includes no correction for cross-shelf transport time.

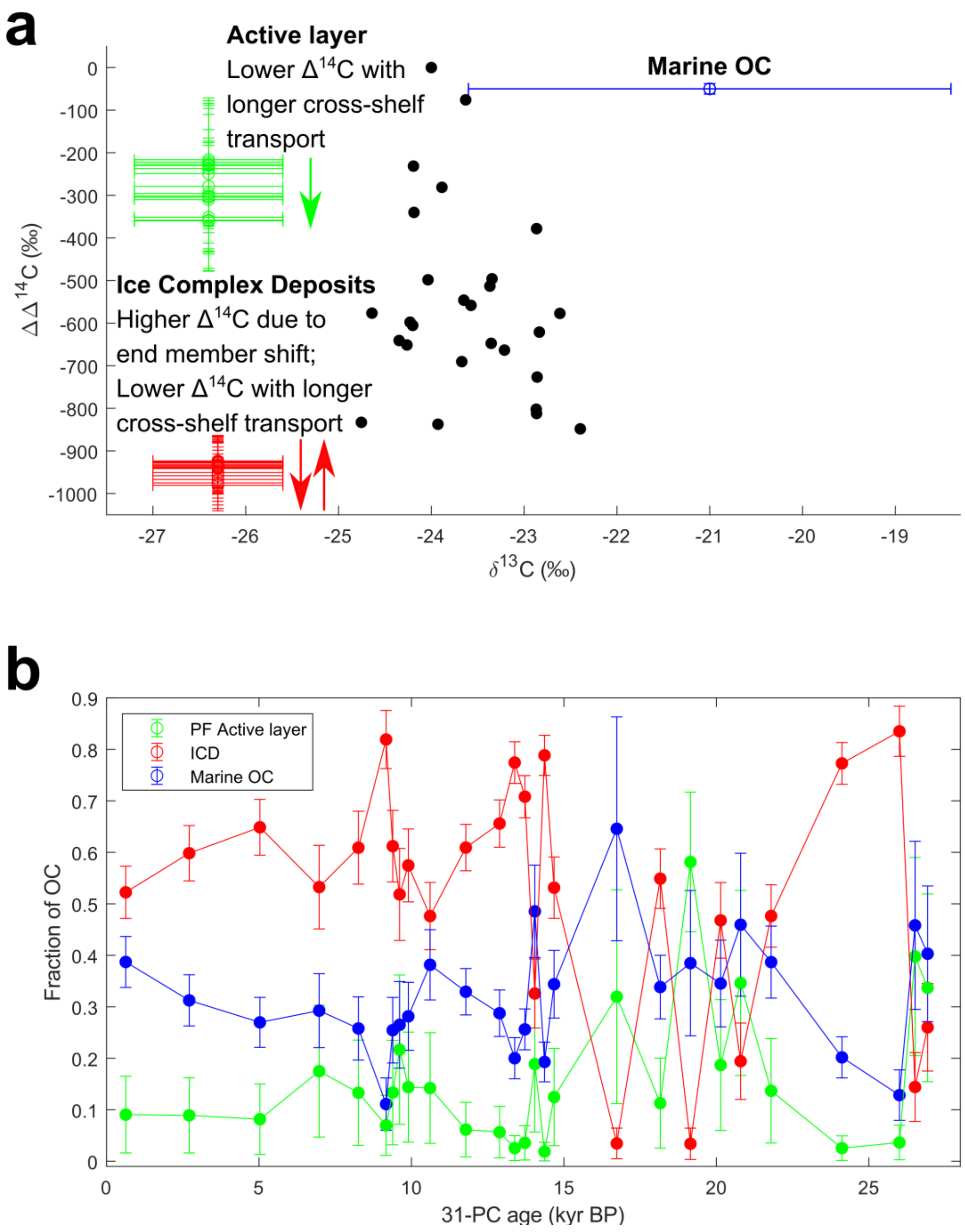

Fig. S3: Input data and results of the source apportionments for samples in the 31-PC. The plots show (a) data of δ^{13}C-OC and the pre-depositional ^{14}C-OC activity ($\Delta\Delta^{14}$C) including the three end members (Permafrost active layer, Ice Complex Deposits and Marine OC). Arrows indicate how the end members vary due to changing cross-shelf transport and relative age of Ice Complex Deposits (Text S1 and S2). Furthermore, (b) the results of the source apportionments as OC fractions of the 31-PC are shown.

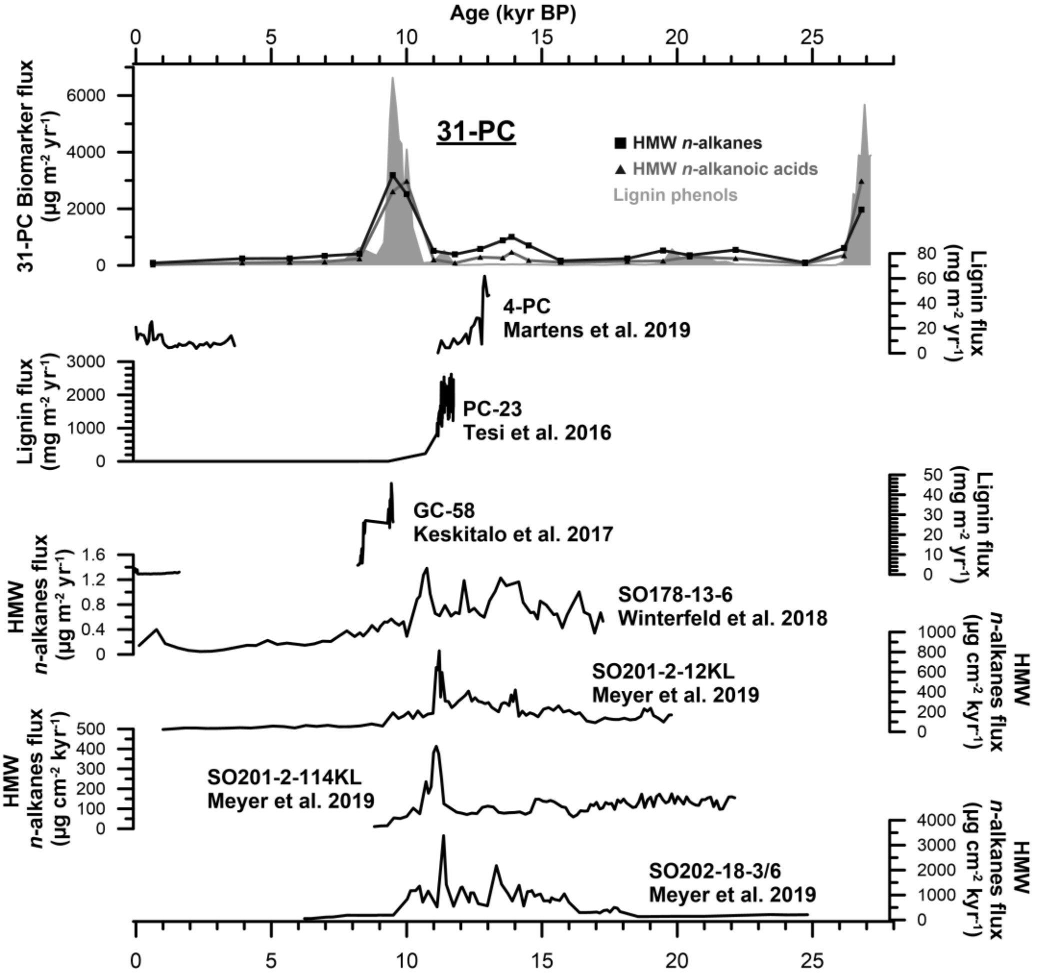

Fig. S4: Comparison of terrigenous biomarker fluxes from the 31-PC with previous studies that reconstructed release of terrigenous OC during the last deglaciation. All compound classes (lignin, *n*-alkanes, *n*-alkanoic acids) are derived by land plants only and allow relative down-core comparison of terrigenous OC input. Lignin fluxes are shown for the 4-PC (*13*), the PC-23 (*11*) and the GC-58 (*17*), which are all located on the East Siberian Arctic Shelf. In addition, the fluxes of high molecular weight (HMW) *n*-alkanes are shown for the core SO178-13-6 (*14*) in the Sea of Okhotsk and the cores SO201-2-12KL, SO201-2-114KL, SO202-18-3/6 from the Bering Sea (*18*). This figure includes no correction for cross-shelf transport time.

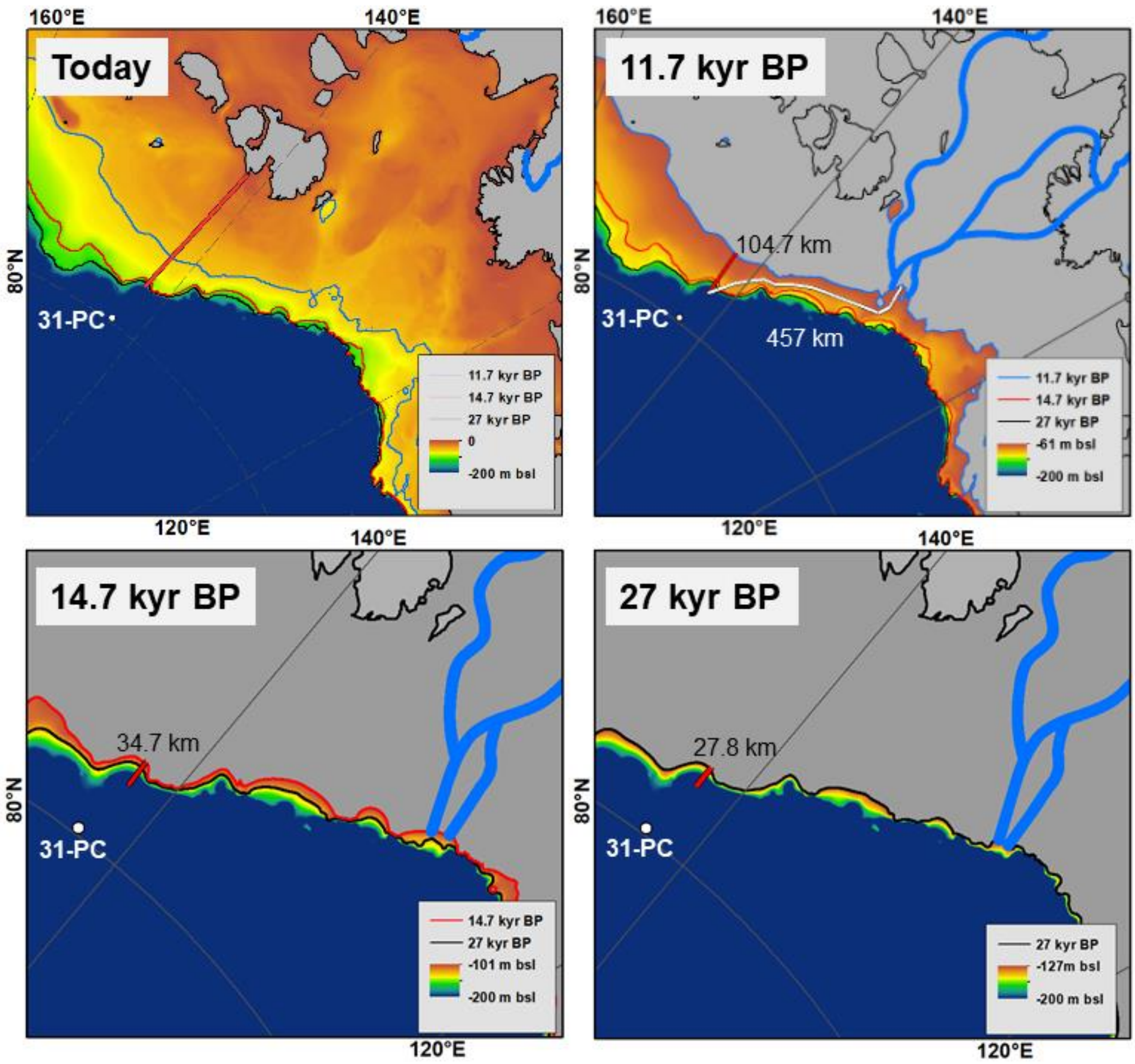

Fig. S5: Assessment of cross-shelf transport between the paleo-coastline and the 31-PC location at modern conditions compared with permafrost carbon remobilization events in the 31-PC. The paleo-coastline was reconstructed using different time steps based on the bathymetry (*31*) reconstructions of the global sea level (*5*). For the time step at 11.7 kyr BP, the red line indicates the route for estimating the minimum cross-shelf transport time while the white line shows the maximum possible cross-shelf transport distance.

Table S1. Total organic carbon content (OC), organic carbon stable isotopes (δ^{13}C-OC), organic carbon radiocarbon activity (Δ^{14}C-OC) and radiocarbon activity corrected for the residence time ($\Delta\Delta^{14}$C-OC) in 31-PC samples.

**Depth** cm	**Age** kyr BP	**OC** %	**δ^{13}C-OC** ‰	**OC flux** g yr^{-1} m^{-2}	**Δ^{14}C-OC** ‰	**$\Delta\Delta^{14}$C-OC** ‰	**AMS label no.**
3	0.3	0.99	-22.6	0.36			
8	0.6	0.86	-22.6	0.38	-611	-577	OS-144952
21	1.5	0.76	-22.8	0.67			
31	2.1	0.75	-23.1	0.59			
41	2.7	0.84	-23.4	0.7	-748	-647	OS-144953
51	3.3	0.76	-23.4	0.67			
60	3.9	0.83	-23.6	0.71			
66	4.3	0.81	-23.7	0.71			
77	5	0.78	-23.7	0.69	-833	-690	OS-147788
87	5.7	0.79	-23.8	0.68			
97	6.3	0.85	-23.7	0.71			
107	7	0.88	-24.2	0.76	-828	-598	OS-144954
117	7.6	0.88	-24.3	0.81			
127	8.3	0.88	-24.3	0.76	-873	-651	OS-144753
137	8.9	0.93	-24.6	0.84			
147	9.2	1.03	-24.8	2.31	-945	-833	OS-147789
157	9.3	0.98	-24.3	5.5			
167	9.4	1.03	-24.3	5.79	-886	-641	OS-144754
177	9.5	0.97	-24.5	5.67			
188	9.6	1.02	-24.6	5.73	-869	-577	OS-144955
198	9.7	0.92	-24.2	5.7			
208	9.8	0.87	-24.1	5.98			
216	9.9	0.86	-24.2	5.97	-882	-605	OS-147790
226	10	0.62	-23.8	3.84			
236	10.2	0.49	-23.4	1.79			
246	10.6	0.49	-23.4	1.07	-866	-513	OS-147791
256	11	0.47	-23.4	1.08			
266	11.4	0.27	-22.9	0.58			
276	11.8	0.38	-22.8	0.93	-910	-621	OS-144956
286	12.1	0.34	-22.6	0.92			
296	12.5	0.35	-22.5	0.91			
306	12.7	0.34	-23.1	1.64			
316	12.9	0.4	-23.2	2.11	-930	-663	OS-147792
326	13.1	0.34	-22.8	1.69			
336	13.2	0.33	-22.7	1.75			
346	13.4	0.29	-22.9	1.51	-963	-812	OS-144755
356	13.6	0.34	-22.8	1.62			
366	13.7	0.4	-22.9	1.94	-948	-727	OS-144961
376	13.9	0.39	-22.8	1.84			
386	14	0.42	-22.9	1.85	-887	-378	OS-144957
396	14.2	0.36	-22.2	1.59			
406	14.4	0.35	-22.4	1.55	-974	-848	OS-144756
416	14.5	0.33	-22.8	1.46			
426	14.7	0.42	-23.7	2.16	-924	-546	OS-147794
436	15.2	0.38	-23.4	0.58			
446	15.7	0.44	-23.7	0.68			
456	16.2	0.47	-23.8	0.77			
466	16.7	0.47	-23.6	0.76	-879	-76	OS-144958
476	17.2	0.47	-24.4	0.81			
486	17.8	0.4	-23.6	0.65			
496	18.2	0.48	-23.6	0.91	-951	-558	OS-144962
506	18.5	0.35	-23.7	0.73			
516	18.8	0.37	-23.9	0.84			
526	19.1	0.45	-24	1.05	-848	0	OS-147795
536	19.5	0.51	-24	1.2			
546	19.8	0.51	-24.2	1.18			
556	20.1	0.48	-24	1.22	-956	-498	OS-144963

566	20.5	0.48	-24.1	1.21			
576	20.8	0.52	-23.9	1.34	-942	-281	OS-144959
586	21.1	0.44	-23.8	1.16			
596	21.5	0.48	-23.8	1.29			
606	21.8	0.42	-23.3	1.04	-964	-496	OS-144757
616	22.2	0.98	-23.8	2.33			
626	23.4	0.44	-23.3	0.34			
636	24.1	0.32	-22.9	0.39	-989	-802	OS-147796
643	24.7	0.36	-22.8	0.37			
653	25.5	0.32	-23.1	0.38			
663	25.7	0.35	-22.9	1.13			
673	25.9	0.38	-22.9	1.75			
683	26	0.58	-23.9	2.78	-993	-837	OS-147797
693	26.2	0.86	-23.9	3.83			
703	26.3	0.87	-24.1	5.11			
713	26.4	0.95	-24.2	5.86			
723	26.5	1.02	-24.2	7.21	-969	-231	OS-144758
733	26.6	0.97	-24.1	6.86			
743	26.7	0.98	-24.3	7.19			
753	26.8	1.04	-24.3	7.51			
763	26.9	1.07	-24.2	6.58	-975	-340	OS-144961
773	27	1.03	-24.3	6.16			
783	27.2	1.07	-24.4	6.88			

Table S2. Lignin data of 31-PC samples. S = syringyl phenols, V = vanillyl phenols, C = cinnamyl phenols, 3,5-Bd = 3,5-Dihydroxybenzoic acid.

Depth<br>cm	Model age<br>kyr BP	Lignin<br>ng m^{-2}	3,5-Bd<br>ng m^{-2}	S/V	C/V	3,5Bd/V	Lignin flux<br>ng yr^{-1} m^{-2}
3	0.3	49	27	0.86	0.17	1.1	65
8	0.6	26	18	2.01	0.27	2.2	46
21	1.5	11	19	1.52	0.16	4.5	43
31	2.1	19	26	1.21	0.14	3.2	62
41	2.7	17	28	0.96	0.14	3.4	68
51	3.3	16	21	0.88	0.18	2.7	66
60	3.9	22	14	0.51	0.2	1.1	75
66	4.3	21	33	0.64	0.16	2.8	78
77	5	23	22	0.52	0.17	1.6	90
87	5.7	36	33	0.55	0.17	1.6	117
97	6.3	63	41	0.49	0.18	1.1	181
107	7	30	56	0.45	0.1	2.9	124
117	7.6	192	57	0.52	0.17	0.5	622
127	8.3	107	29	0.52	0.17	0.5	345
137	8.9	245	59	0.52	0.18	0.4	804
147	9.2	259	68	0.54	0.18	0.5	2273
157	9.3	250	60	0.52	0.17	0.4	5164
167	9.4	293	67	0.53	0.19	0.4	6626
177	9.5	241	72	0.48	0.15	0.5	5612
188	9.6	201	62	0.53	0.17	0.5	4437
198	9.7	202	63	0.5	0.16	0.5	4290
208	9.8	99	27	0.47	0.14	0.4	2275
216	9.9	138	44	0.54	0.17	0.6	4094
226	10	68	32	0.54	0.13	0.8	1330
236	10.2	5.9	21	0.87	0.14	7.1	79
246	10.6	19	18	0.53	0.04	1.5	128
256	11	67	21	0.36	0.07	0.5	523
266	11.4	1.4	4.5	0.94	0	6.5	13
276	11.8	1.5	7.9	2.03	0.79	20	16
286	12.1	2.2	5.7	1.07	0	5.5	25
296	12.5	2.1	4.8	0.86	0	4.2	22
306	12.7	1.6	5.1	0.26	0	4.1	33
316	12.9	1.8	7.7	1.22	0.14	10	42
326	13.1	1.9	4.7	0.51	0	3.7	39
336	13.2	1.7	4.4	1.28	0	6.0	40
346	13.4	1.3	4.1	0.8	0	5.8	29
356	13.6	1.6	3.9	0.97	0	4.7	35
366	13.7	2.0	4.2	1.2	0	4.6	39
376	13.9	1.1	4.5	0.34	0.07	5.7	38
386	14	1.4	7.5	5.48	0	34	30
396	14.2	2.0	5.3	0.73	0	4.6	40
406	14.4	1.4	4.1	2.29	0	9.8	27
416	14.5	1.7	5.1	2.5	0	11	29
426	14.7	1.2	7.1	2.69	0	22	24
436	15.2	1.9	6.4	1.05	0.09	7.3	11
446	15.7	3.3	7.1	1.37	0	5.2	20
456	16.2	4.4	9.6	0.65	0.15	3.9	28
466	16.7	2.4	7.4	1.15	0.08	6.8	16
476	17.2	4.6	9.8	0.43	0.02	3.1	32
486	17.8	6.9	10	0.69	0.16	2.8	54
496	18.2	4.0	8.6	0.75	0.17	4.1	33
506	18.5	1.8	4.4	0.65	0.14	4.3	24
516	18.8	4.0	12	0.49	0.16	4.8	45
526	19.1	11	18	0.33	0.18	2.5	106
536	19.5	53	30	0.29	0.12	0.8	573
546	19.8	21	22	0.33	0.19	1.6	243
556	20.1	21	23	0.34	0.18	1.7	222
566	20.5	21	20	0.3	0.12	1.3	239

576	20.8	19	20	0.32	0.16	1.5	256
586	21.1	11	10	0.29	0.15	1.4	122
596	21.5	9.8	16	0.38	0.2	2.6	134
606	21.8	3.3	10	0.63	0.14	5.4	37
616	22.2	2.0	7.9	1.92	0	12	15
626	23.4	2.6	9.7	0.4	0.01	5.3	7
636	24.1	1.9	8.7	1.39	0	11	7
643	24.7	2.6	8.7	1.36	0.14	8.4	9
653	25.5	1.1	7.3	1.83	0	18	7
663	25.7	0.8	7.0	0	0	0.0	12
673	25.9	2.5	12	1.18	0.27	12	64
683	26	6.0	27	0.5	0.21	7.7	128
693	26.2	42	41	0.37	0.25	1.6	869
703	26.3	41	29	0.39	0.22	1.1	1135
713	26.4	83	49	0.37	0.18	0.9	2527
723	26.5	74	37	0.41	0.16	0.8	2453
733	26.6	113	63	0.38	0.15	0.9	3895
743	26.7	119	61	0.42	0.18	0.8	3840
753	26.8	174	75	0.41	0.15	0.7	5676
763	26.9	125	52	0.4	0.15	0.6	3821
773	27	130	56	0.4	0.17	0.7	3887
783	27.2	167	71	0.42	0.17	0.7	5375

Table S3. Concentrations and fluxes of high molecular weight (HMW) *n*-alkanes and *n*-alkanoic acids in 31-PC, as well the ratio of HMW to low molecular weight (LMW; C_{15}, C_{17}, C_{19}) *n*-alkanes, and the three degradation proxies carbon preference index (CPI; for both compound classes) and ratio of high molecular weight (HMW) *n*-alkanoic acids to *n*-alkanes.

**Depth**	**Model age**	**HMW *n*-alkanes**	**HMW/LMW *n*-alkanes**	**CPI_{alk}**	**HMW *n*-alkanes flux**	**HMW *n*-alkanoic acids**	**CPI_{acid}**	**HMW *n*-alkanoic acids flux**	**HMW *n*-alkanoic acids / *n*-alkanes**
cm	kyr BP	ng m^{-2}			ng yr^{-1} m^{-2}	ng m^{-2}		ng yr^{-1} m^{-2}	
3	0.3	65	14	4.5	85	19	2.2	25	0.29
51	3.3	59	4.9	3.5	240	22	2.2	88	0.37
77	5	62	13	4.1	245	29	2.2	114	0.46
97	6.3	116	4.3	3.2	335	44	2.3	127	0.38
117	7.6	126	15	4.6	409	74	2.7	238	0.58
167	9.4	141	27	4.2	3186	115	2.5	2609	0.82
216	9.9	85	16	4.5	2514	100	2.7	2978	1.18
246	10.6	75	10	4.1	511	30	2.1	205	0.40
266	11.4	42	2.2	2.0	390	9.1	2.7	84	0.21
296	12.5	57	21	4.0	581	29	1.8	291	0.50
346	13.4	39	37	5.4	881	12	1.8	263	0.30
366	13.7	52	40	3.2	1005	24	1.7	465	0.46
406	14.4	37	4.9	3.2	704	9.0	1.5	174	0.25
436	15.2	28	24	3.2	162	19	1.4	108	0.67
486	17.8	31	40	5.3	241	19	2.1	143	0.59
526	19.1	52	28	3.7	526	16	1.9	157	0.30
556	20.1	34	13	4.0	360	29	2.1	302	0.84
606	21.8	50	3.0	1.9	546	22	2.0	242	0.44
636	24.1	25	475	2.3	94	17	2.1	66	0.70
683	26	29	16	4.5	613	16	2.2	347	0.57
743	26.7	61	23	4.0	1967	92	3.3	2977	1.51

Table S4. Source fractions of active layer (AL), Ice Complex Deposits (ICD) and marine organic carbon (MOC) based on carbon isotopes ($\delta^{13}C$, $\Delta\Delta^{14}C$), calculated using the Markov chain Monte Carlo model.

Depth	Model age	Active layer		ICD		MOC	
cm	kyr BP	Mean	s.d.	Mean	s.d.	Mean	s.d.
8	0.6	0.09	0.07	0.52	0.05	0.39	0.05
41	2.7	0.09	0.07	0.60	0.05	0.31	0.05
77	5.0	0.08	0.07	0.65	0.05	0.27	0.05
107	7.0	0.17	0.13	0.53	0.08	0.29	0.07
127	8.3	0.13	0.10	0.61	0.07	0.26	0.06
147	9.2	0.07	0.06	0.82	0.06	0.11	0.05
167	9.4	0.13	0.10	0.61	0.07	0.25	0.06
188	9.6	0.22	0.15	0.52	0.09	0.27	0.08
216	9.9	0.14	0.11	0.57	0.07	0.28	0.07
246	10.6	0.14	0.11	0.48	0.07	0.38	0.07
276	11.8	0.06	0.05	0.61	0.05	0.33	0.05
316	12.9	0.06	0.05	0.66	0.05	0.29	0.05
346	13.4	0.03	0.02	0.77	0.04	0.20	0.04
366	13.7	0.04	0.03	0.71	0.04	0.26	0.04
386	14.0	0.19	0.13	0.33	0.07	0.49	0.09
406	14.4	0.02	0.02	0.79	0.04	0.19	0.04
426	14.7	0.12	0.09	0.53	0.06	0.34	0.07
466	16.7	0.32	0.21	0.03	0.03	0.65	0.22
496	18.2	0.11	0.09	0.55	0.06	0.34	0.06
526	19.1	0.58	0.14	0.03	0.03	0.38	0.14
556	20.1	0.19	0.13	0.47	0.07	0.35	0.08
576	20.8	0.35	0.18	0.19	0.07	0.46	0.14
606	21.8	0.14	0.10	0.48	0.06	0.39	0.07
636	24.1	0.03	0.02	0.77	0.04	0.20	0.04
683	26.0	0.04	0.03	0.84	0.05	0.13	0.05
723	26.5	0.40	0.19	0.14	0.07	0.46	0.16
763	26.9	0.34	0.18	0.26	0.08	0.40	0.13

Table S5. Concentrations of individual *n*-alkanes and *n*-alkanoic acids in samples from the 31-PC normalized to surface area (SA)

Depth	Age	Alk-C15	Alk-C16	Alk-C17	Alk-C18	Alk-C19	Alk-C20	Alk-C21	Alk-C22	Alk-C23	Alk-C24	Alk-C25	Alk-C26	Alk-C27	Alk-C28	Alk-C29	Alk-C30	Alk-C31	Alk-C32	Alk-C33
cm	kyr BP	ng m^{-2}	ng m^{-2}	ng m^{-2}	ng m^{-2}	ng m^{-2}	ng m^{-2}	ng m^{-2}	ng m^{-2}	ng m^{-2}	ng m^{-2}	ng m^{-2}	ng m^{-2}	ng m^{-2}	ng m^{-2}	ng m^{-2}	ng m^{-2}	ng m^{-2}	ng m^{-2}	ng m^{-2}
3	0.3	0.8	0.7	1.0	2.3	1.0	1.4	2.4	1.8	4.7	2.6	6.9	3.0	12	2.6	13	1.8	13	1.0	3.3
51	3.3	1.9	2.0	2.2	2.4	2.5	2.6	3.3	3.0	5.4	2.6	5.0	1.6	8.1	1.1	12	2.9	12	2.8	4.8
77	5.0	0.9	0.2	0.9	1.2	1.1	1.3	2.1	2.2	5.1	2.5	7.0	3.2	11	2.7	12	1.5	12	1.2	3.3
97	6.3	4.2	4.6	4.8	5.1	5.4	5.7	7.0	6.0	11	5.0	10	3.4	16	2.5	23	6.2	23	5.9	10
117	7.6	1.0	1.0	1.8	2.6	2.1	2.3	4.7	3.8	10	5.4	14	6.0	23	4.7	25	3.1	25	1.6	7.9
167	9.4	0.3	0.5	1.0	1.2	1.7	2.0	4.5	4.5	12	9.2	18	6.6	27	5.0	28	2.7	25	1.2	6.6
216	9.9	0.5	0.4	0.9	1.2	1.5	1.5	3.4	3.1	8.0	4.6	10	4.0	15	3.0	16	1.9	16	0.8	4.4
246	10.6	0.9	1.1	1.7	2.9	1.8	1.9	3.1	2.4	5.2	2.7	7.5	4.1	13	3.1	16	2.3	15	1.7	4.2
266	11.4	2.7	2.7	2.9	3.0	3.0	3.1	3.3	2.9	4.2	1.9	3.3	1.5	4.1	1.1	7.4	3.6	7.1	3.8	4.4
296	12.5	0.6	0.2	0.3	0.6	0.6	0.7	1.4	1.9	4.5	3.0	7.1	3.4	9.3	2.5	12	1.4	11	0.4	2.2
346	13.4	0.0	0.1	0.4	1.3	0.3	0.4	0.9	0.8	2.2	0.8	3.8	2.1	6.8	1.5	8.7	1.0	9.1	0.4	2.2
366	13.7	0.0	0.3	0.3	0.5	0.5	0.8	0.9	1.3	2.6	4.6	4.4	2.4	8.1	2.3	11	1.5	11	0.9	2.7
406	14.4	1.2	1.4	1.5	2.3	1.3	1.4	1.6	1.2	2.4	0.0	2.9	2.7	5.5	2.0	7.0	1.9	7.5	1.8	2.9
436	15.2	0.0	0.0	0.2	0.5	0.4	0.6	0.6	0.7	1.6	3.1	2.3	1.4	5.4	0.9	5.1	0.5	5.5	0.3	1.5
486	17.8	0.1	0.0	0.0	0.6	0.4	0.7	0.8	1.0	1.9	1.1	2.8	1.5	4.9	1.4	7.0	0.6	8.3	0.1	1.7
526	19.1	0.0	0.0	0.2	0.4	0.8	1.0	2.0	1.9	4.1	4.2	5.7	2.2	10	2.1	10	1.3	10	0.5	2.6
556	20.1	0.2	0.3	0.5	0.8	0.9	1.1	1.8	1.6	3.1	1.5	3.8	1.8	5.8	1.7	6.6	0.8	7.1	0.4	1.5
606	21.8	2.4	2.2	2.3	2.7	2.5	2.5	2.9	2.7	3.9	3.8	4.4	3.2	6.3	3.3	7.3	3.2	7.8	2.6	3.8
636	24.1	0.0	0.4	0.0	0.8	0.0	0.9	0.3	1.1	1.1	1.3	1.5	1.8	3.2	1.8	4.9	1.4	5.8	0.7	0.9
683	26.0	0.4	0.2	0.1	0.5	0.6	0.6	0.7	0.9	2.2	1.2	3.4	1.5	5.2	1.2	6.1	0.7	5.9	0.3	1.2
743	26.7	0.0	0.3	0.4	0.9	1.0	1.3	2.2	2.5	6.1	3.8	7.7	3.0	11	2.6	12	1.3	11	0.5	2.7

Depth	Age	Acid-C14	Acid-C15	Acid-C16	Acid-C17	Acid-C18	Acid-C19	Acid-C20	Acid-C21	Acid-C22	Acid-C23	Acid-C24	Acid-C25	Acid-C26	Acid-C27	Acid-C28	Acid-C29	Acid-C30	Acid-C31	Acid-C32
cm	kyr BP	ng m^{-2}	ng m^{-2}	ng m^{-2}	ng m^{-2}	ng m^{-2}	ng m^{-2}	ng m^{-2}	ng m^{-2}	ng m^{-2}	ng m^{-2}	ng m^{-2}	ng m^{-2}	ng m^{-2}	ng m^{-2}	ng m^{-2}	ng m^{-2}	ng m^{-2}	ng m^{-2}	ng m^{-2}
3	0.3	10	4.4	51	4.8	51	0.7	5.2	2.6	5.1	2.2	4.7	2.7	4.8	1.4	2.6	0.8	1.2	0.5	0.0
51	3.3	4.3	2.0	41	2.4	39	0.0	3.0	1.7	3.9	2.2	5.0	2.1	4.2	1.9	3.6	1.2	2.1	0.8	0.8
77	5.0	6.2	2.5	40	3.6	44	0.0	3.8	2.1	4.4	2.6	6.2	3.6	7.4	1.7	3.4	1.5	2.6	1.3	1.0
97	6.3	8.8	4.4	79	5.0	77	0.0	6.2	3.6	7.8	4.2	10	3.9	8.3	3.5	7.2	2.4	4.3	2.5	1.6
117	7.6	10	4.8	40	5.7	41	0.6	7.4	4.0	11	5.5	17	8.4	20	4.6	11	2.9	5.0	2.4	2.2
167	9.4	25	10	103	11	104	0.0	12	6.7	16	10	27	14	33	6.3	14	4.8	8.3	4.0	3.5
216	9.9	8	4.0	37	4.2	37	0.0	5.3	3.4	8.6	5.5	17	10	28	6.1	15	5.2	10	3.2	4.7
246	10.6	12	4.9	62	4.8	65	0.0	4.1	2.3	4.7	2.5	5.3	3.6	6.6	2.2	4.6	1.8	2.8	1.2	2.2
266	11.4	5.1	2.2	44	2.0	43	0.0	1.7	1.0	1.8	1.2	2.1	1.1	1.6	0.8	1.4	0.0	1.3	0.0	0.7
296	12.5	6.3	2.1	50	2.3	52	0.0	2.5	1.8	3.6	2.8	4.9	4.2	6.5	2.2	3.3	1.8	3.1	1.1	1.3
346	13.4	7.9	2.5	45	3.1	50	0.0	2.1	1.1	2.0	1.1	2.0	1.6	2.3	0.8	1.4	0.8	0.8	0.0	1.7
366	13.7	14	3.8	73	4.7	82	0.0	3.6	1.9	4.3	2.6	4.9	3.8	5.9	1.7	2.8	1.3	1.9	1.7	0.0
406	14.4	5.9	1.9	32	2.2	36	0.0	1.8	0.8	1.7	0.8	1.6	1.2	1.6	0.6	1.0	0.8	0.6	0.5	1.2
436	15.2	14	4.0	66	5.3	76	0.0	2.9	1.8	3.0	2.0	3.5	2.8	4.2	1.2	2.0	2.0	1.4	1.4	0.0
486	17.8	5.7	2.1	35	2.3	37	0.0	2.2	1.5	2.7	1.9	3.7	2.8	4.7	1.3	2.2	1.0	1.9	0.0	0.9
526	19.1	10	3.8	46	4.1	49	0.0	2.8	1.7	3.0	1.7	3.4	2.1	3.8	1.0	1.9	0.9	1.3	1.3	0.0
556	20.1	7.2	2.8	41	2.9	44	0.0	2.4	1.7	3.4	2.3	5.0	3.6	6.6	1.9	3.5	1.7	3.4	1.1	1.6
606	21.8	8.6	2.6	48	3.1	54	0.0	2.2	1.3	2.5	1.7	3.7	3.0	5.2	1.5	2.4	1.0	2.3	1.0	1.9
636	24.1	6.2	1.7	58	2.6	64	0.0	2.5	0.9	2.1	1.3	2.9	2.5	4.3	1.4	2.2	0.9	1.9	0.0	0.9
683	26.0	4.9	1.8	31	2.0	34	0.0	1.8	1.1	2.2	1.4	3.6	2.2	4.3	0.9	1.6	0.7	1.4	0.6	0.9
743	26.7	6.8	3.3	38	3.6	36	0.8	3.5	2.1	5.1	3.2	14	7.8	29	4.7	15	4.7	10	2.8	4.6
